# Novel immune cross-talk between inflammatory bowel disease and IgA nephropathy

**DOI:** 10.1080/0886022X.2024.2337288

**Published:** 2024-04-17

**Authors:** Qianqian Yan, Zihao Zhao, Dongwei Liu, Jia Li, Shaokang Pan, Jiayu Duan, Zhangsuo Liu

**Affiliations:** a Department of Integrated Traditional and Western Nephrology, the First Affiliated Hospital of Zhengzhou University, Zhengzhou, P. R. China; b Research Institute of Nephrology, Zhengzhou University, Zhengzhou, P. R. China; c Henan Province Research Center for Kidney Disease, Zhengzhou, P. R. China; d Key Laboratory of Precision Diagnosis and Treatment for Chronic Kidney Disease in Henan Province, Zhengzhou, P. R. China

**Keywords:** Immunoglobulin a nephropathy, inflammatory bowel disease, cross-talk, FDX1

## Abstract

The mechanisms underlying the complex correlation between immunoglobulin A nephropathy (IgAN) and inflammatory bowel disease (IBD) remain unclear. This study aimed to identify the optimal cross-talk genes, potential pathways, and mutual immune-infiltrating microenvironments between IBD and IgAN to elucidate the linkage between patients with IBD and IgAN. The IgAN and IBD datasets were obtained from the Gene Expression Omnibus (GEO). Three algorithms, CIBERSORTx, ssGSEA, and xCell, were used to evaluate the similarities in the infiltrating microenvironment between the two diseases. Weighted gene co-expression network analysis (WGCNA) was implemented in the IBD dataset to identify the major immune infiltration modules, and the Boruta algorithm, RFE algorithm, and LASSO regression were applied to filter the cross-talk genes. Next, multiple machine learning models were applied to confirm the optimal cross-talk genes. Finally, the relevant findings were validated using histology and immunohistochemistry analysis of IBD mice. Immune infiltration analysis showed no significant differences between IBD and IgAN samples in most immune cells. The three algorithms identified 10 diagnostic genes, MAPK3, NFKB1, FDX1, EPHX2, SYNPO, KDF1, METTL7A, RIDA, HSDL2, and RIPK2; FDX1 and NFKB1 were enhanced in the kidney of IBD mice. Kyoto Encyclopedia of Genes and Genomes analysis showed 15 mutual pathways between the two diseases, with lipid metabolism playing a vital role in the cross-talk. Our findings offer insights into the shared immune mechanisms of IgAN and IBD. These common pathways, diagnostic cross-talk genes, and cell-mediated abnormal immunity may inform further experimental studies.

## Introduction

Immunoglobulin A nephropathy (IgAN), the most common glomerulonephritis worldwide and a leading cause of chronic kidney disease, affects all age groups [1–3]. The mucosal immune system-kidney axis, initially studied by Heather Reich, Jennifer Gommerman, and KeiHaniuda, has been a focus in IgAN research [4]. Inflammatory bowel disease (IBD), comprising ulcerative colitis and Crohn’s disease, results from an unbalanced relationship between genes, the environment, microbes, and innate immunity [5–7]. IgAN is the most common renal complication when performing renal biopsy [8–10].

Similar to IgAN with unclear etiology and pathogenesis, IBD is a troublesome heterogeneous disease with an unignorable influence on millions of people worldwide [11–13]. A genome-wide study revealed that a multitude of loci associated with IgAN were related to immune-mediated IBD, intestinal barrier preservation, and gut pathogens reactions [14]. Similarly, a prominent British epidemiological study showed that patients with IBD are more susceptible to renal diseases [15]. In most conditions, not merely did the break out of glomerulonephritis accordant with acute deterioration of intestinal inflammation, but renal function was modified by treating the gastrointestinal disorder occurrence [16–18]. Furthermore, gut-relevant lymphoid tissue is hyper-reactive in patients with IgAN, and the gut-renal link is a novel treatment option for patients with IgAN [19–21]. Forty years ago, glomerular diseases were proposed as extraintestinal manifestations (EIM) of IBD [22]; therefore, the association between IgAN and IBD warrants clinical attention.

Given the complexity and incomplete understanding of IgAN and IBD, immune correlation and potential inflammatory cross-talk remain unknown. We hypothesized the existence of cross-talk between IBD and IgAN at the transcriptomic level. Bioinformatics involves the integration and analysis of biological data using multiple methods. Rapid advancements in bioinformatics have enabled the comprehension of the pathological and molecular mechanisms of diseases and implementation of precision medicine [23]. Because of the dilemma and inadequacy of biomarkers to explore the probability of disease occurrence and acquisition, we present a series of bioinformatics analyses to systematically demonstrate the potential cross-talk between genes and common immune microenvironments and uncover the immunocorrelation contributing to the pathogenesis of IgAN and IBD. The 10 hub genes identified in this study offer novel perspectives for further research into the comorbidity of IBD and IgAN.

## Materials and methods

### Study design and data

**Figure 1.**
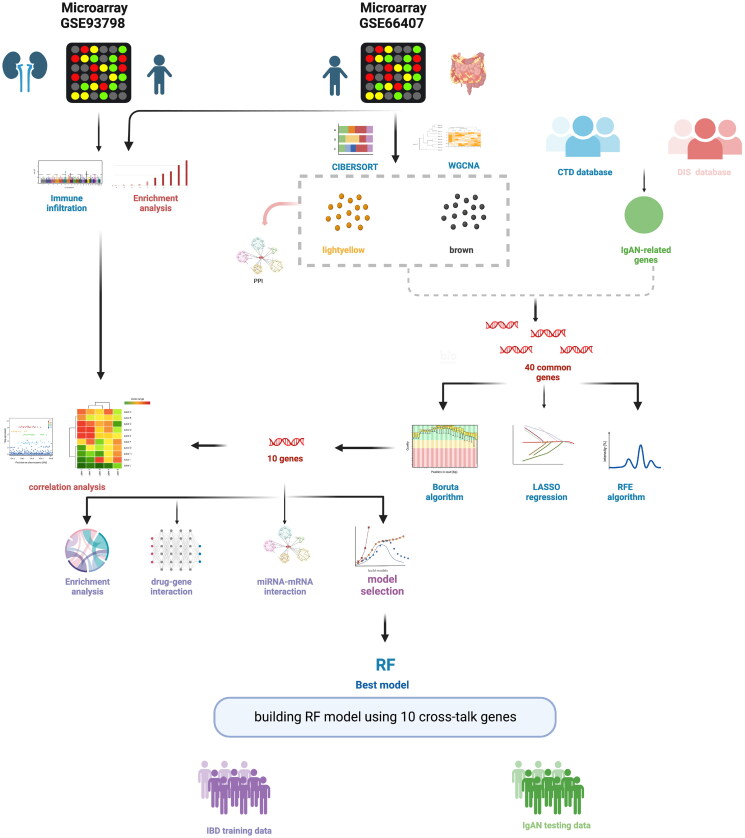
Workflow of this study. All datasets were extracted from Gene Expression Omnibus (GEO) database. The LASSO regression, RFE algorithm, and Boruta algorithm were used to select the optional cross-talk genes. RF model was considered as the best model to predict IgAN using the 10 cross-talk genes. In addition, protein–protein interaction (PPI) and functional enrichment analysis of the DEGs were performed. Graphic created with BioRender.com.

We used the keywords ‘Inflammatory bowel disease’ and ‘IgA nephropathy’ to retrieve IBD and IgAN gene expression profiles in the Gene Expression Omnibus (GEO) database. The eligibility criteria were as follows: 1. Datasets including the IBD and IgAN sequencing results from the analysis of human tissue biopsies and blood samples. 2. Datasets including controls and cases, with no fewer than 20 samples in each group to reduce errors caused by a small sample size. 3. IBD samples without IgAN, and *vice versa*. Finally, the GEO datasets GSE66407, GSE93798, GSE186507, and GSE193677 were selected. GSE66407 includes 368 samples (264 samples with Crohn’s disease or ulcerative colitis and 99 samples from healthy tissues; five patients with an unconfirmed diagnosis were excluded). Additional details of the datasets are presented in Supplementary Tables 1 and 2. GSE93798 includes 42 samples that were confirmed *via* renal biopsy, including 20 samples with IgA nephropathy and 22 healthy control tissue samples. GSE186507 comprises 821 blood samples from patients with IBD and 209 samples from healthy controls. GSE193677 contains 2029 biopsy specimens from patients with IBD and 461 samples from healthy controls. All definitively diagnosed samples from the four datasets were included. Raw data were rectified and quantile-normalized. A schematic representation of the study is presented in Figure 1. All methods were performed in accordance with relevant guidelines and regulations.

### Exploration of immune characteristics in IBD and IgAN patients

The gene set variation analysis (GSVA) algorithm was applied to compute the pathway activation score [24]. For understanding and interpreting synergetic pathway-level variation in transcriptomics experiments represented under diverse circumstances, the gene set enrichment analysis (GSEA) was applied to identify whether there were statistically significant deviations between the two groups [25]. The pathway-level alteration for all disease-related genes in IBD and IgAN was examined to determine whether there was a reduplicative pathway.

### Immune microenvironment analysis

The CIBERSORTx method was employed to calculate cell composition according to expression profiles. The deconvolution algorithm assessed the cellular abundance and cell type-specific gene expression patterns [26,27].

Based on the expression levels of genes in 28 published gene sets for immune cells, single-sample gene set enrichment analysis (ssGSEA) was applied to compute the extent of infiltration of 28 immune cell types [28]. Immune, microenvironment, and stroma scores were calculated using xCell, which transforms gene expression profiles into enrichment scores [29]. Based on the deconvolution algorithm, the xCell algorithm integrated the strengths of gene enrichment analysis and can assess the abundance of immune cells.

### Weighted gene co-expression network analysis

The clusters or modules of highly correlated genes can be recognized by weighted gene co-expression network analysis (WGCNA), a practical network-based approach. WGCNA was conducted on gene expression data of IBD. Gene modules that distinguish the pathways or functions of subtypes ground on gene profiles employing the WGCNA [30]. In our research, the soft threshold *β* was 12 in the WGCNA of IBD. WGCNA was employed on the IBD dataset to detect vital modules associated with immune infiltration. Correlations between different modules and the proportion of 22 immune cells were analyzed using Pearson’s correlation. The modules that linked IBD and its immune microenvironment were identified and regarded as vital.

### Identification of potential cross-talk genes

IgAN-related genes were downloaded from the Comparative Toxicogenomics Database (CTD; http://ctdbase.org/) and DisGeNET database (https://www.disgenet.org/home/). The top two significant modules associated with T Cell, B Cells, and Macrophages were considered IBD-related modules. We identified the potential cross-talk genes by overlapping the IBD-related genes with IgAN-related genes.

### Identification of optimal diagnostic cross-talk genes

Least absolute shrinkage and selection operator (LASSO) regression, recursive feature elimination (RFE) algorithm, and Boruta algorithm were performed in the R project to further screen the risk cross-talk genes between IBD and IgAN. We collated the R packages used frequently in the Supplementary Table 3. The combination of the support vector machine (SVM) model, RF model, and GLM allowed us to identify the most appropriate model based on the optimal diagnostic cross-talk gene expression on the IBD dataset. These machine learning algorithms are mature and commonly used methods for screening key genes, and each algorithm involves different principles [31–34]. The diagnosis of IBD or not was considered the response variable, and IBD-related genes were expected as explanatory variables.

### Random forest modeling employing optimal cross-talk genes

The selected cross-talk genes’ expression values that form the IBD gene expression profile were extracted. Then to further validate the diagnostic utility of these cross-talk genes, the RF model with the gene expression data and sample types was established. The gene expression data of the selected optimal features constituted the IBD gene expression profile. The RF models with the gene expression data and sample diagnosis were established. The IBD dataset was imported as training data, and the IgAN dataset was input as testing data. We choose the accuracy rate of the test set as the prediction effectiveness. We then found an external validation set with a large amount of data, including tissue samples (GSE193677) and blood samples (GSE186507), to verify the predictive power of the RF model built by the 10 cross-talk genes.

### Infiltrating immune cells interact with diagnostic cross-talk genes

To inspect the correlation between infiltrating immune cells and diagnostic cross-talk genes, we performed spearman’s rank correlation test in R. Correlations were illustrated using the ‘ggplot2’ package. Subsequently the correlation among immune infiltration, calculated using different algorithms, was determined *via* Spearman correlation analysis.

To probe the underlying mechanism of diagnostic gene cross-talk in IBD and IgAN, we used a single-gene GSEA algorithm for the two diseases, and the cutoff threshold was established at *p* < 0.05.

### Transcription factor-regulated and pathway analysis of the cross-talk genes

Transcription factors are proteins that attach to specific genes and control their rate of transcription [35]. The transcription factors responsible for the cross-talk of genes should be identified to comprehend gene regulatory networks. Therefore, we used bioinformatics to identify the TFs that determine the cross-talk between IBD and IgAN. A transcription factor enrichment tool, ChIP-X Enrichment Analysis 3, arranges the TFs of the submitted gene sets [36]. A TF-target regulatory mutual effect network database, TRRUST, was recognized *via* the manual curation of Medline abstracts [37]. We uploaded IBD-relevant genes to the website and obtained the IBD-related TFs. The TFs common in the two websites were selected. Based on the TF-target relationship, we sought out IBD-related pairs and established a TF-target gene mutual effect network using the Cytoscape software.

We screened the remarkably enriched pathways of IBD-related genes to identify the activated pathways. Potential cross-talk pathways that may link IBD and IgAN and the genes functioning in each pathway were identified. Finally, we established a pathway-gene interaction network.

### Identification of miRNAs interacting with cross-talk genes

Generally, mRNA expression is regulated by microRNAs (miRNAs). Because of their impact on gene expression, stable existence in body tissues and fluids, and feasible use as disease biomarkers, miRNAs have become a hotspot of basic and translational biomedical research. Accordingly, relative miRNA expression is crucial in disease progression [38]. In addition, miRNAs with targeted gene interactions were incorporated to track the miRNAs that attach to gene transcripts and adversely affect protein expression [39]. Exploring potential regulatory miRNAs may provide new insights into disease treatment. We used the miRNA-target tool ENCORI (https://starbase.sysu.edu.cn/) to investigate the miRNAs of the cross-talk genes. The data in the ENCORI database is from CLIP-seq and miRNA-target interactions illustrated by predicting the mutual effect of miRNA targets on the binding sites of Ago proteins. This database contains the results of seven prediction programs (PITA, RNA22, miRmap, DIANA-microT, miRanda, PicTar, and TargetScan). This method assists in screening the top miRNAs with high degrees and exploring their biological functions and features, resulting in a valid biological hypothesis.

mRNAs regulate multiple critical biological processes, and their functions are associated with cellular localization. The Human Protein Atlas (HPA: https://www.proteinatlas.org/) database was used to detect the cellular localization of these cross-talk genes.

### Drug–gene interaction network

The drug–gene interaction database (DGIdb) was used to detect the underlying drugs interacting with the 10 diagnostic cross-talk genes MAPK3, NFKB1, FDX1, RIPK2, EPHX2, HSDL2, RIDA, SYNPO, and KDF1. The CYTOSCAPE software was used to visualize the drug–gene network [40].

### DSS-induced mouse IBD model

Ten male C57BL/6 mice (6 weeks old, 20 g) were purchased from Gem Pharmatech Co., Ltd. (Nanjing, China) and randomly divided into two groups (*n* = 5). The mice were fed under SPF conditions at the Experimental Animals Center of Zhengzhou University (Zhengzhou, Henan, China). All animal care and experiments in this study were approved by the Welfare and Ethical Committee for Experimental Animal Care of the Zhengzhou University of Medicine. All animal experiments were performed according to the ARRIVE guidelines for reporting animal research [41]. All procedures were performed according to the relevant guidelines and regulations. After two weeks of adaptive feeding, a chemically induced IBD mouse group was established by administering the C57BL/6 mice with 3.5% DSS for 7 days (a very mature method to develop a ulcerative colitis mouse model) [42, 43]. The control mice were maintained in the same environment and fed pure water. After 7 days, the drinking water of the IBD mice was changed to pure water, and the mice in both the groups were sacrificed on Day 8. Mice were anesthetized with 1% sodium (50 mg/kg), and the kidney tissue was collected.

### Histology and immunohistochemistry

Kidney sections were fixed in 4% paraformaldehyde and stained using periodic acid-Schiff (PAS), hematoxylin–eosin (H&E), and Masson’s trichrome. We employed a microwave-based antigen retrieval technique to perform immunohistochemistry in 4-um paraffin kidney sections. The prepared tissue sections were incubated overnight at 4 °C with primary antibodies against FDX1 (1:100, 12592-1-ap, rabbit polyclonal, Proteintech, Wuhan, China), MAPK3 (1:100, A16686, rabbit polyclonal, ABclinal Technology, Wuhan, China), and NFKB1(1:100, A6667, rabbit polyclonal, ABclinal Technology, Wuhan, China). The sections were then washed and incubated with a secondary antibody at room temperature for 1 h. Finally, the sections were visualized using an HRP–DAB system (Proteintech, Wuhan, China) and stained with hematoxylin to detect HRP activity. The sections were observed under an Olympus BX53F microscope.

### Immunoblot assay

Total protein from the kidney tissue was extracted using radioimmunoprecipitation assay buffer (RIPA buffer, R0010, Solarbio, Beijing, China). The protein concentrations were collected after centrifugation at 12,000 rpm at 4 °C for 10 min and quantified using a BCA kit (PC0020, Solarbio, Beijing, China). Subsequently, the proteins were boiled with SDS sample buffer at 100 °C for 10 min and electrophoresed with 10% or 15% SDS-PAGE gels. The proteins were then subjected to western blotting [44]. Primary antibodies against FDX1 (1:1000, 12592-1-ap) and GAPDH (1:1000, 10494-1-AP) were purchased from Proteintech (Wuhan, China), and NFKB1 (1:1000, A6667) was obtained from ABclinal Technology (Wuhan, China). Secondary antibodies (1:5000) were purchased from Proteintech (Wuhan, China).

## Results

### Immune characteristics and pathway of IBD and IgAN

We executed GSEA on every dataset and compared the similar enrichment pathway between IBD and IgAN datasets. Each disease’s DEGs were included in the GSEA employing gene set ‘c5.all.v7.5.1.entrez’. A total of 493 mutual pathways were confirmed in IgAN and IBD. We confirmed many significant pathways related to immunityin IgAN and IBD datasets, including leukocyte-mediated immunity and B cell-mediated immunity (Supplementary Figure 1A, B).

**Figure 2.**
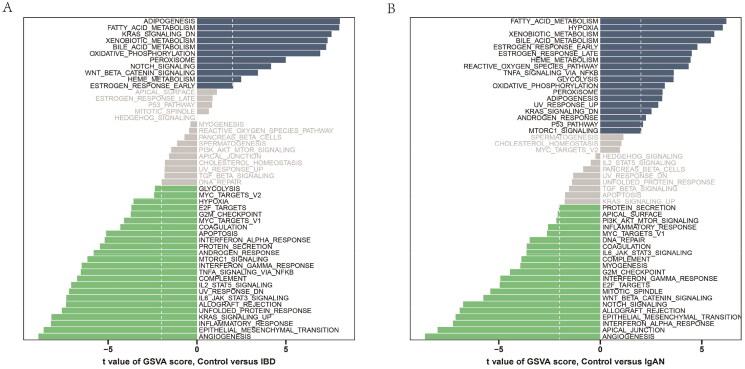
The underlying immune characteristics diversity in IgAN and IBD samples. Differences in pathway activities scored per sample by GSVA in IgAN vs. healthy controls and IBD samples vs. healthy controls. (A) The differences of the Reactome pathway enrichment score between IBD and healthy controls. (B) The differences of the Reactome pathway enrichment score between IgAN and healthy controls.

Compared to the healthy controls, more enriched pathways were discovered in IBD patients, such as ADIPOGENESIS, FATTY_ACID_METABOLISM, KRAS_STGNALING_DN (Figure 2A). The enrichment pathways mentioned above were also exhibited in IgAN patients (Figure 2B). Hence, we continued to use the GSVA algorithm to compare the two diseases, and no significant enrichment pathway was found (Supplementary Figure 2).

### Immune microenvironment analysis

CIBERSORTx, ssGSEA, and xCell were performed to illustrate the immunological function’s comparability better. We explored the comparability in immune infiltration between IBD samples and IgAN samples in 22 subpopulations of immune cells by using the CIBERSORTx algorithm. Figure 3A shows the results acquired from IBD and IgAN patients. According to Figure 3B, there were no prominent diversities between IBD and IgAN samples in the majority of immune cells, including macrophage M1, which were deemed pro-inflammatory and promoted inflammation [45].ssGSEA showed that the expression levels of 28 immune cell subtypes were not significantly different between IBD and IgAN (Figure 3C). On the left side of Supplementary Figure 3A is a cluster diagram of IBD and IgAN patients, showing that most samples were mixed. The proportion of the 11 significant cells is displayed on the right side of the figure, indicating that the distribution ratio of these 11 immune cells was similar between the two diseases. Sixty-four kinds of cells had no significant expression difference between these two groups in xCell analysis (Supplementary Figure 3B). Immune scores, microenvironment scores, and stroma scores were also found to have no significant difference between the two diseases (Supplementary Figure 3C). The consequences of the three algorithms mentioned above reveal that the two diseases are probably in a parallel immune infiltration environment.

Figure 3. Relationship between immune microenvironment analysis. Comparison of immune characteristics between IBD and IgAN. (A) An intuitive picture of the percentage of the 22 immune cells in IBD and IgAN patients. (B) The proportion of 22 immune cells in two diseases. (C) The expression of 28 immune cells in IBD patients and IgAN patients. (*p < 0.05, **p < 0.01; Mann–Whitney *U* test). ns, not significant. Pairwise comparisons of the immune microenvironment are shown, with a color gradient denoting Spearman’s correlation coefficients. Edge width represents Mantel’s *r* statistic for corresponding distance correlations, and edge color corresponds to statistical significance.

**Figure:**
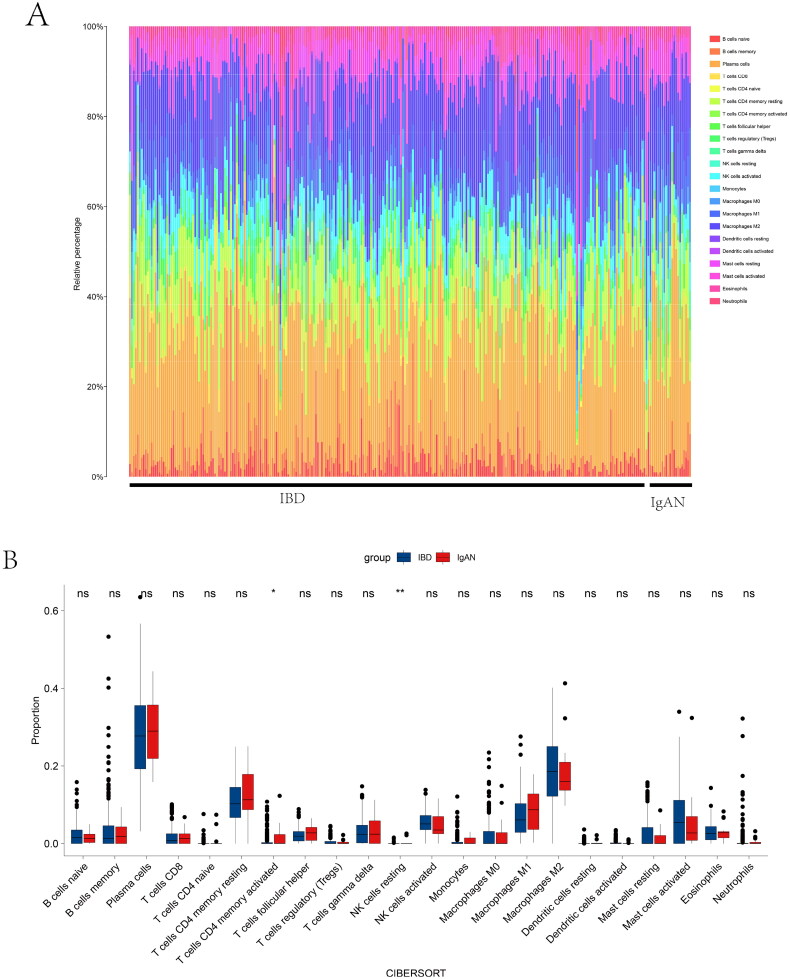

**Figure:**
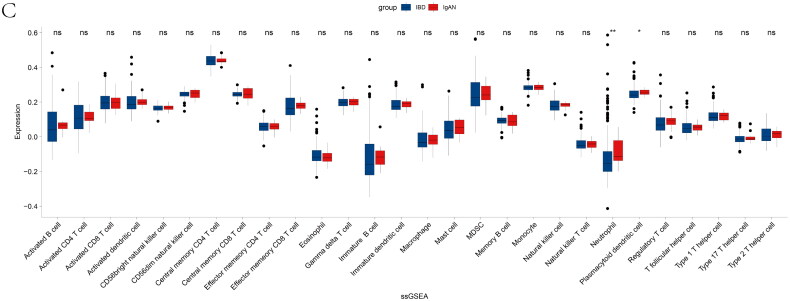

### IBD-related gene screening using weighted gene co-expression network analysis

Two outliers (GSM1621605 and GSM1621589) were eliminated from the following study because they were detected during sample clustering (Supplementary Figure 4A, B). Subsequently, 0.9 was set as the scale-free fit index to acquire an optimal soft threshold of 12 for founding the scale-free network (Supplementary Figure 4A). On the basis of dynamic tree cutting, the genes were converged into numerous modules based on hierarchical clustering and merged when setting the threshold to 0.25 (Supplementary Figure 4D). The top two immune infiltration-related modules, the MElightyellow and MEbrown modules, were most associated with various T cells, B cells, and macrophages (Figure 4A, B, Supplementary Figure 4E). We then obtained IBD-related genes by merging genes from the MElightyellow with the MEbrown module, getting 699 genes in total. Finally, the IBD-related genes intersected with IgAN-related genes, yielding 40 potential cross-talk genes.

Figure 4. Identification of IBD-related genes associated with immune infiltration and functional enrichment analysis. (A) Clustering dendrograms for IBD-related genes. (B) Correlation heatmap between module eigen genes and immune infiltration cells. (C) Heatmap of enriched ontology clusters. (D) Enriched ontology cluster colored according to cluster ID. (E) PPI MCODE components. C–E were created by Metascape (http://metascape.org).

**Figure:**
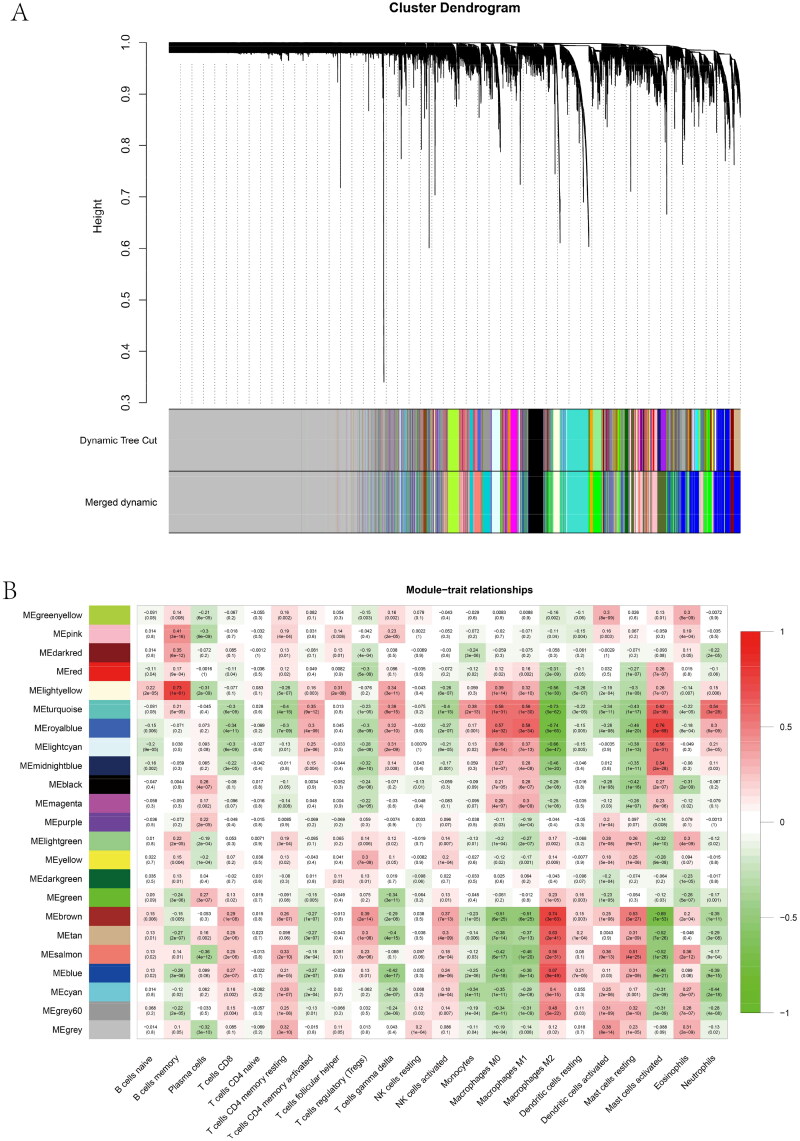

**Figure:**
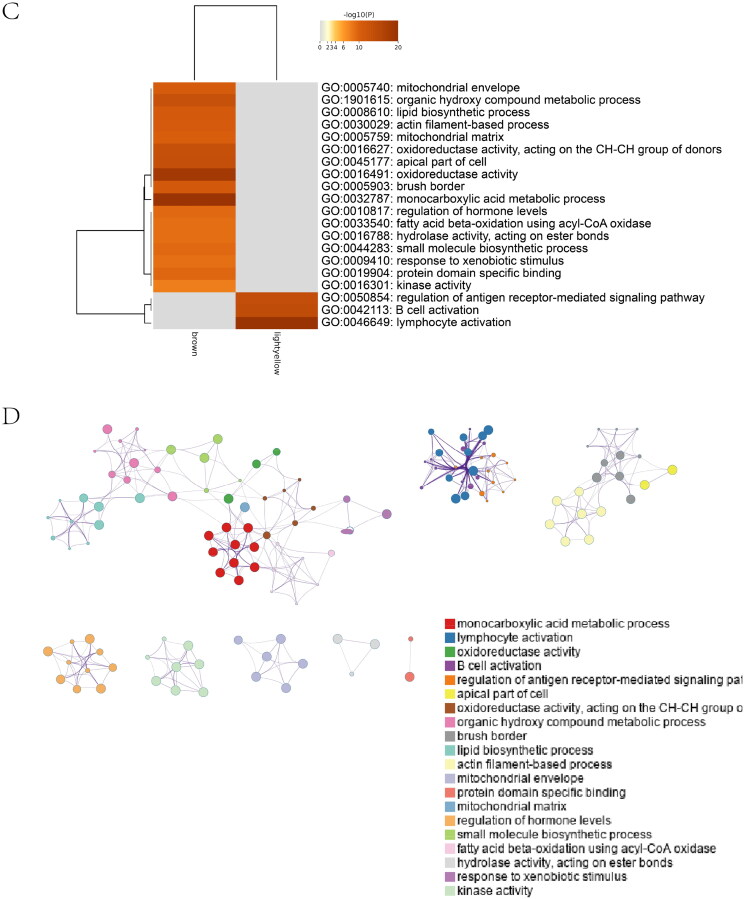

**Figure:**
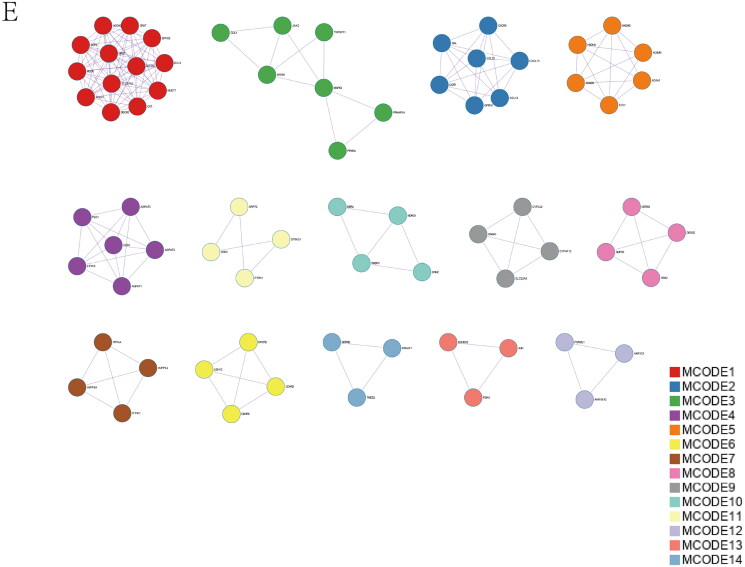

### Comparison of the top two modules gene enrichment analysis

The top 20 GO pathways were exhibited *via* heatmap, some of which were consistent with GSVA results in these two important modules: lipid biosynthetic process, oxidoreductase activity, and fatty acid beta-oxidation using acyl-CoA oxidase (Figure 4C). A series of GO pathways were polymerized, and a network was established on the basis of Kappa-statistical similarities, each color on behalf of a specific cluster identity, each circle node on behalf of a particular term of the pathway, and the size of nodes fits the number of input genes proportionally (Figure 4D). We used the Molecular Complex Detection (MCODE) algorithm to recognize intensively linked network neighborhoods. A specific color represents the respective MCODE network (Figure 4E).

### Prediction of optimal cross-talk genes, construction of the protein–protein interaction network, and building the machine learning model

The heatmap of shared genes between two diseases is shown in Supplementary Figure 5A, B. The Go analysis discovered that mutual genes are consumingly connected with the MAPK family pathway, including regulation of the JNK cascade, stress-activated MAPK cascade, and stress-activated MAPK cascade (Figure 5A). The established PPI network of mutual genes involved 39 nodes and 114 edges (Figure 5B). According to MCODE, a plug-in of Cytoscape (Cytoscape_v3.8.0), the score of the most crucial module was 5.091 (Figure 5C). The hub genes were confirmed by CytoHubba (Figure 5D). MAPK3 may be the essential gene that links IgAN and IBD because of its highest score in the biological network (Supplementary Table 4).

Figure 5. Expression level of common genes. (A) Gene Ontology pathway enrichment analysis of the 40 common genes. (B) The PPI network analysis of the 40 common genes. (C) The most vital module (score = 5.091) was identified by MCODE. (D) The hub genes identified by CytoHubba.

**Figure:**
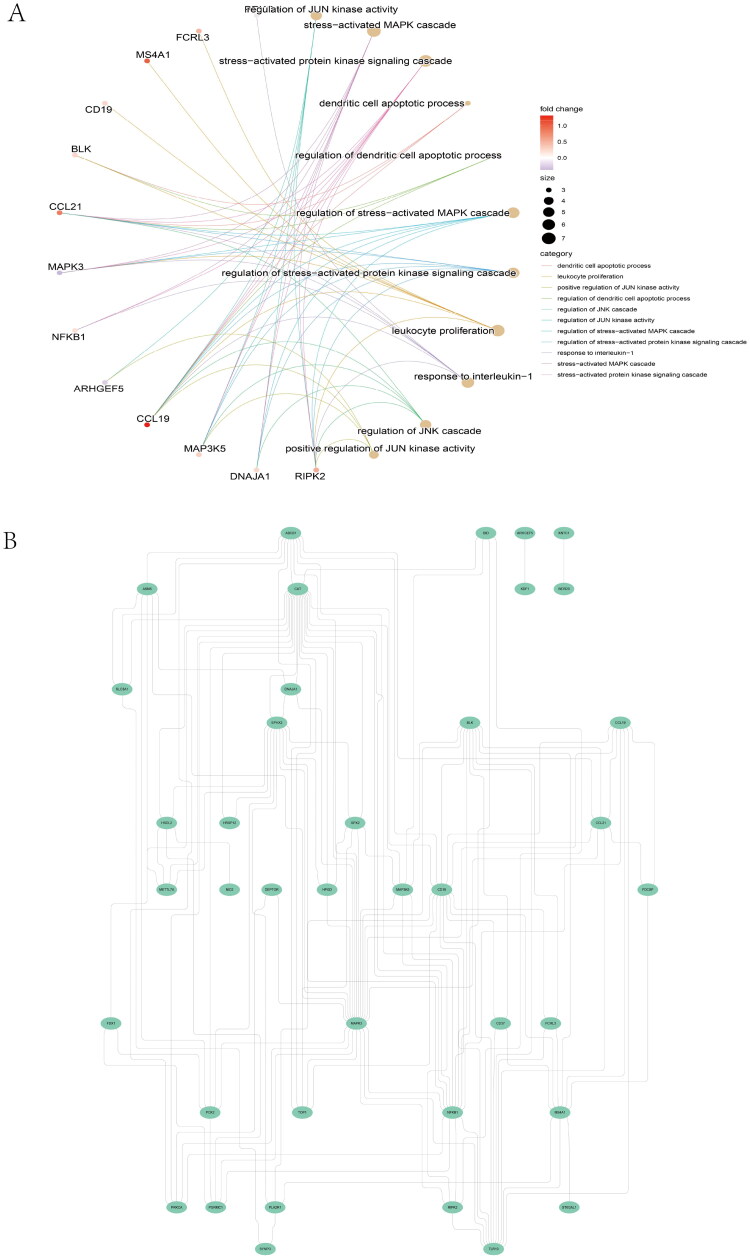

**Figure:**
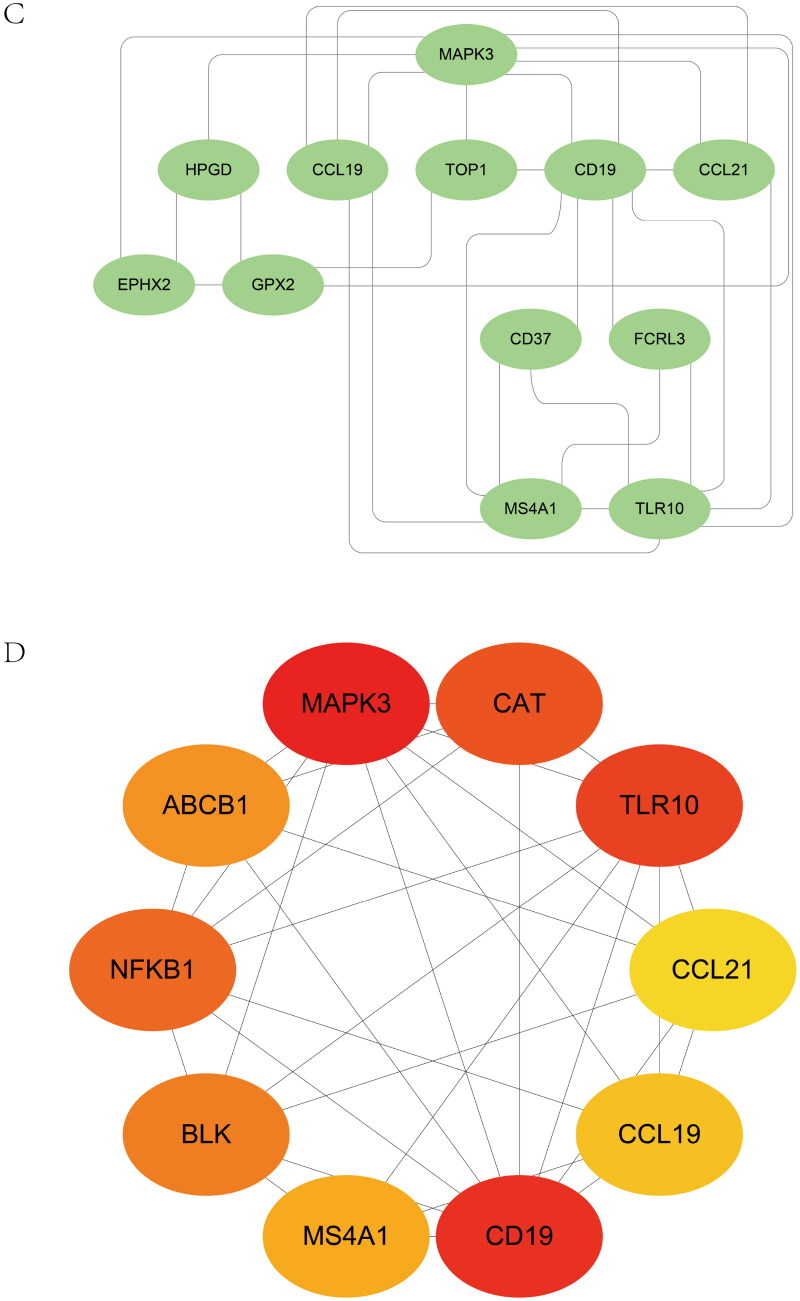

Optimal cross-talk genes were identified with the Boruta algorithm, REF algorithm, and LASSO regression (Figure 6A–D). A total of 10 genes were finally selected by overlapping genes derived from the three algorithms (Supplementary Table 5). Figure 6E shows the importance of the top 20 genes in the Boruta algorithm. The expression levels of genes selected by the Boruta algorithm in IBD patients and healthy controls are shown in Supplementary Figure 5C.

Figure 6. Feature and model selection. Cross-talk gene selection *via* Boruta algorithm (A), LASSO regression (B, C), and RFE algorithm (D). (A) The yellow box indicates the 29 confirmed genes. (B) By applying the LASSO model to identify the optimal genes, the partial likelihood deviance curve was plotted vs. log(lambda). Ground on 1 SE of the minimum criteria (the 1-SE criteria) to draw dotted vertical lines. (C) Confirmed 27 genes with non-zero coefficients by optimal lambda. (D) Sixteen feature genes were selected by using the RFE algorithm. The abscissa of the figure is the variable of the number of genes, and the ordinate is the exact value of the whole dataset gauged under the variable. The results indicate that the score is high when the minimum variable is 16, which means that 16 features might map the entire dataset. RFE, recursive feature elimination. (E) Importance ranking based on the Boruta algorithm. (F) Accumulated residual distribution map of the sample. (G) Boxplot of the residuals of the sample. The root means square of the residuals was shown by a red dot. (H) The importance of the variables in the three models. (I) A correlation heatmap illustrates the relationships among immune cell infiltration and 10 cross-talk genes (absolute abundance, CIBERSORTx algorithm) in IgAN and IBD samples. Among all cases, the cells, M2 macrophages, neutrophils, and activated mast cells are highly related to cross-talk genes. (J) The correlation heat map of CIBERSORTx and xCell. M2 macrophages and neutrophils were the most associated with cross-talk genes. (K) A correlation heatmap between CIBERSORTx and ssGSEA. Neutrophils, Macrophages M0, Macrophages M1, activated Mast cells, and memory B cells were demonstrated to have positively correlated with almost 28 kinds of cells of ssGSEA algorithm. (L) A correlation heatmap between CIBERSORTx and 10 cross-talk genes. Macrophages M2 are strongly associated with nearly these 10 genes.

**Figure:**
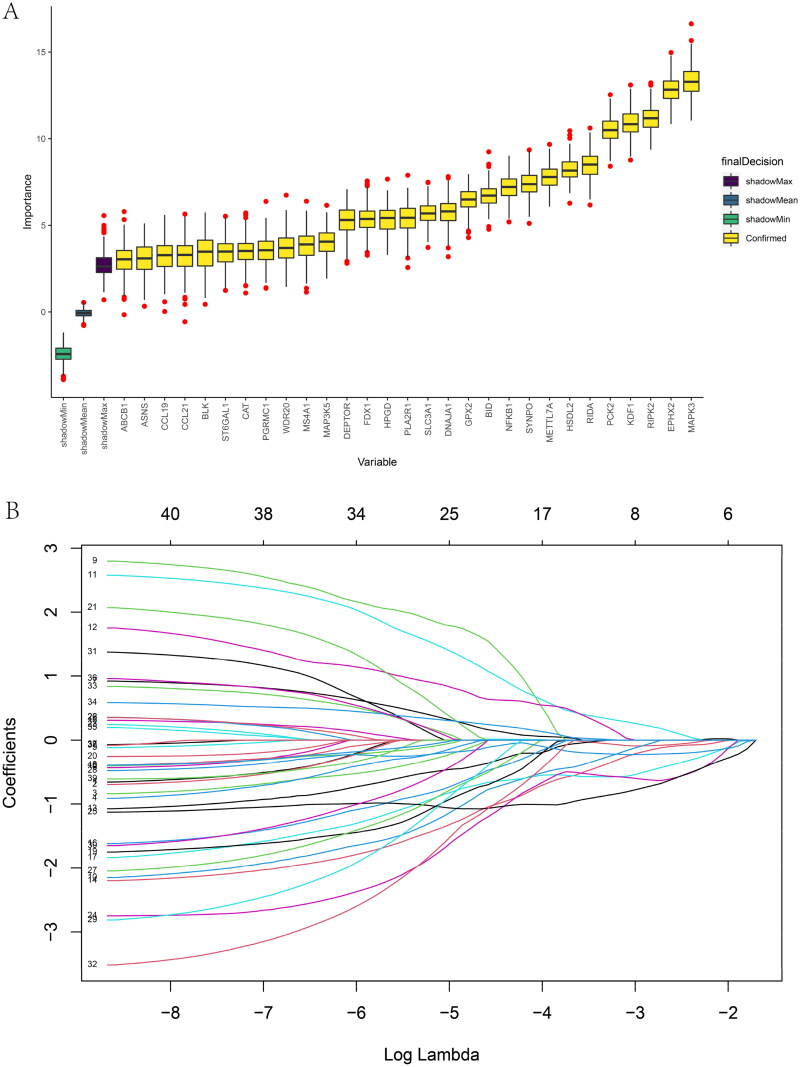

**Figure:**
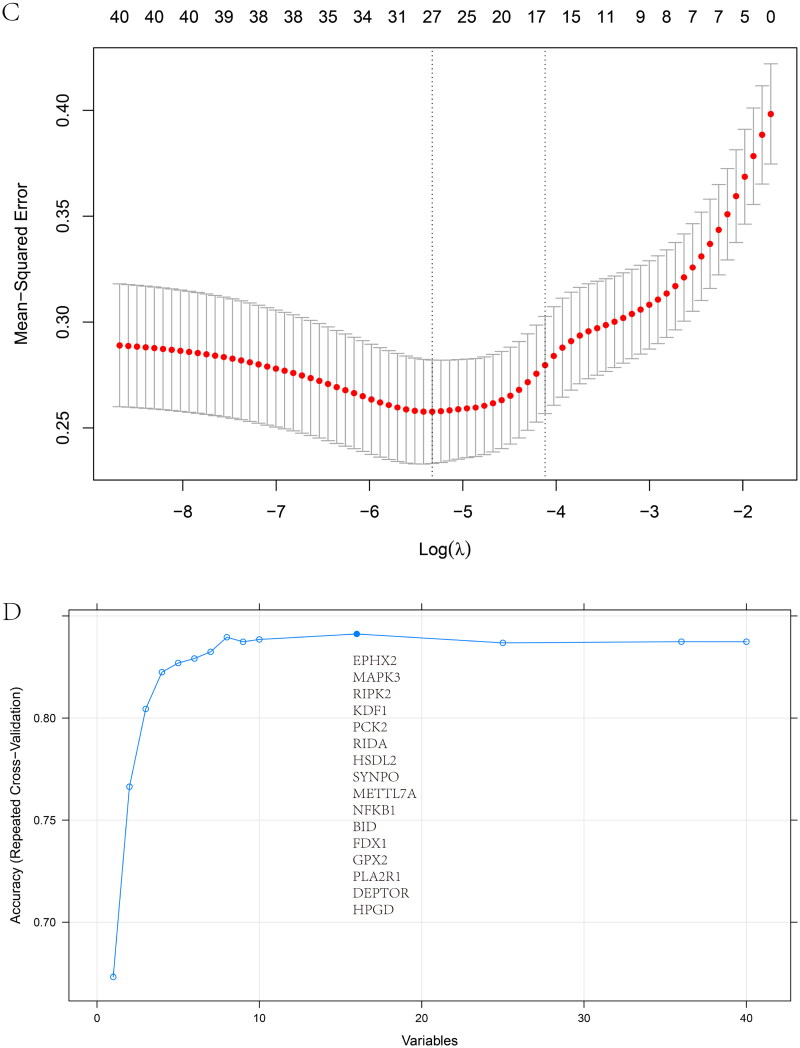

**Figure:**
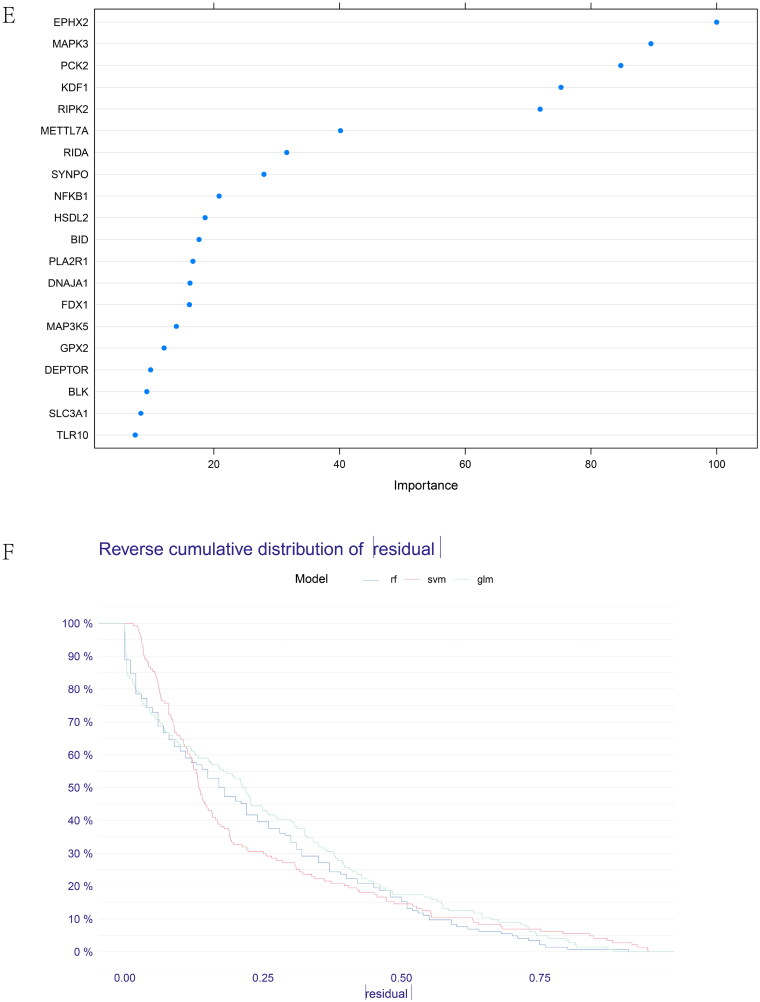

**Figure:**
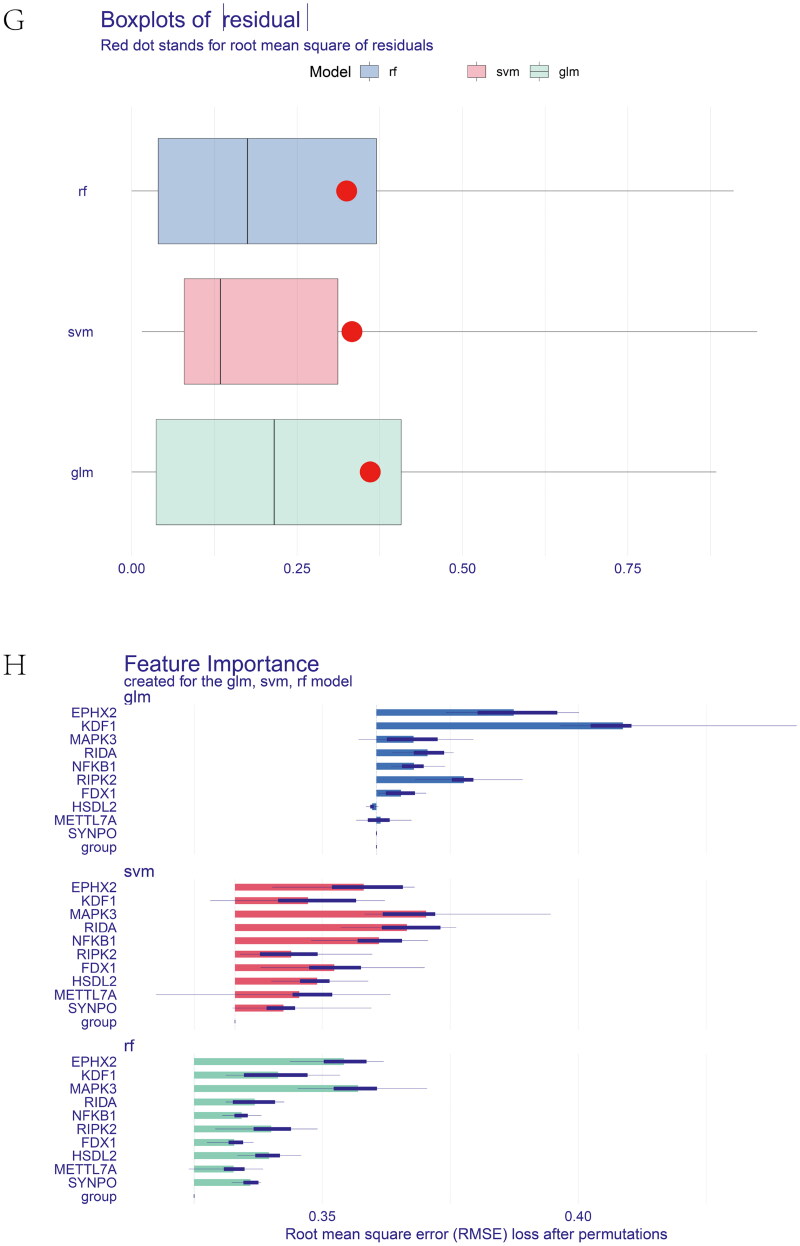

**Figure:**
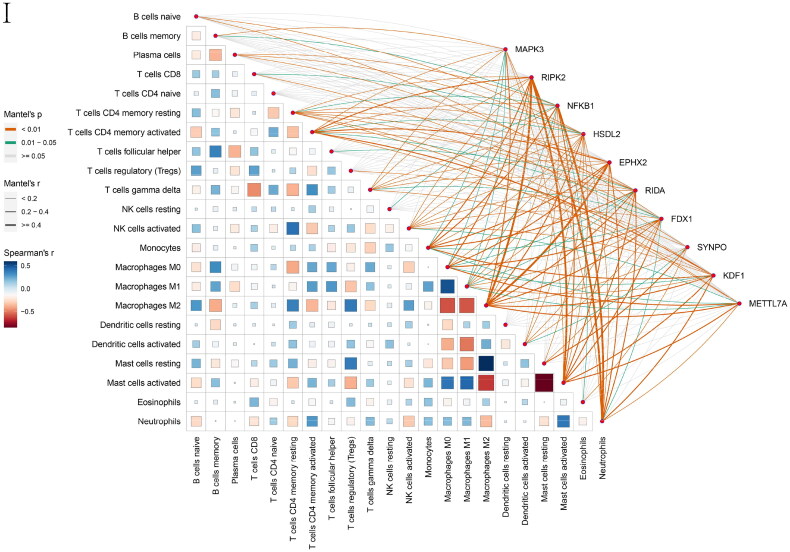

**Figure:**
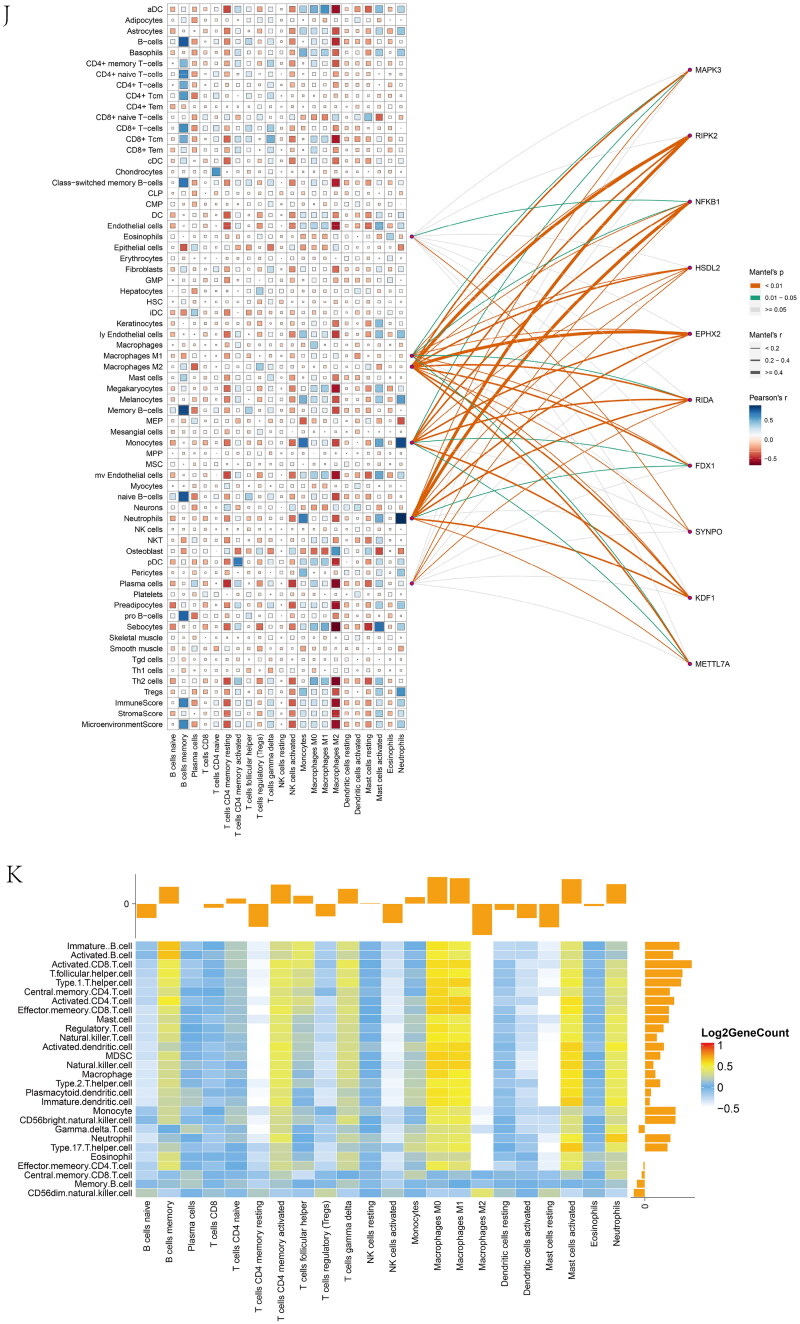

**Figure:**
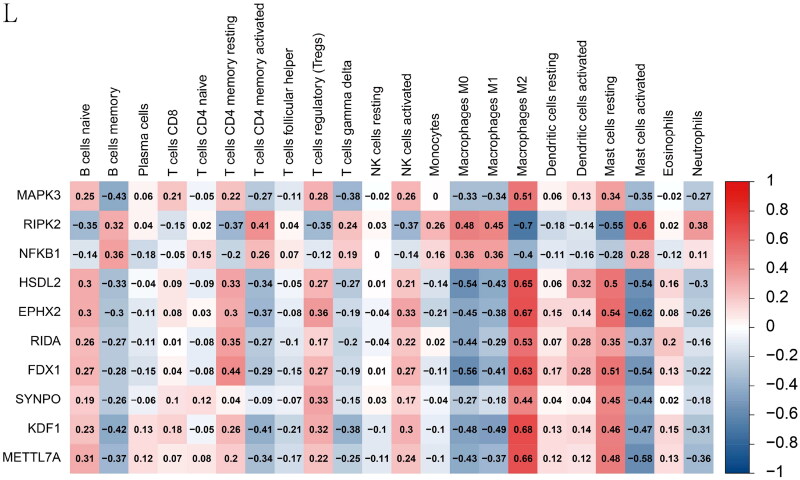

Three models, containing RE, SVM, and GLM, were built on the basis of the IBD dataset to pick out and create the optimal prediction model. As shown in Figure 6F–H, the best suitable model was the RF model because of its minor sample residual. The diagnostic accuracy of the cross-talk genes was computed by receiver-operating characteristic (ROC) analysis. The area under the curve (AUC) training and testing data values were 100% and 80.1%, respectively (Supplementary Figure 5D). ROC curve analysis verified the diagnostic efficacy of the model created using the IBD dataset for IgAN and the predictive accuracy of the cross-talk genes, confirming these diagnostic cross-talk genes associated with IBD and IgAN. To date, no biomarkers in clinical use allow physicians to explore the correlation between the two diseases. These genes act as a link between the two diseases. When IBD patients have abnormal cross-talk gene expression, physicians need to be aware that patients are at potential risk for IgAN. Good diagnostic models also seem to predict that patients can be spared the pain of biopsy in the near future. Table 1 shows the predicted outcome of the RF model. Supplementary Figure 5E exhibits the importance ranking of 10 genes in the RF model. The expression of the 10 diagnostic cross-talk genes in the patients with the two diseases is shown in Supplementary Figure 5F, G, respectively.

**Table 1.** The prediction of RF model in IgAN (GSE93798).

IgAN	RF predict IBD	RF predict non-IBD
Inflammatory bowel disease	30	42
Healthy controls	108	566

Tables 2 and 3 show the prediction of 10 cross-talk genes for the validation set. The prediction accuracy was 80% (GSE193677) and 77% (GSE186507), respectively. The results indicated that our model established by 10 cross-talk genes had excellent predictive power, even in the blood samples.

**Table 2.** The prediction of RF model in IBD (tissue samples GSE193677).

IBD	RF predict IBD	RF predict non-IBD
IgA nephropathy	13	1
Healthy controls	9	19

**Table 3.** The prediction of RF model in IBD (blood samples GSE186507).

IBD	RF predict IBD	RF predict non-IBD
Inflammatory bowel disease	1	42
Healthy controls	61	566

### Relationship between immune microenvironment and cross-talk genes

The Mantel test was performed to decipher the drivers of the relationships between the immune microenvironment and 10 cross-talk genes (Figure 6I). Macrophage M2 and neutrophils were responsible for all closely related cross-talk genes. A similar result was observed in the correlation heat map of CIBERSORTx and xCell (Figure 6J). We further explored the relationship between the ssGSEA and CIBERSORTx algorithms. The ssGSEA algorithm showed that neutrophils, macrophage M0, and macrophage M1 were positively correlated with approximately 28 cell types (Figure 6K).

Macrophage M2 was strongly associated with the 10 genes (Figure 6L). NFKB1 and RIPK2 were significantly negatively correlated with macrophage M2. In contrast, the other eight genes were significantly positively correlated with macrophage M2. These results indicate that macrophages and neutrophils play critical roles in IgAN and IBD.

### Transcription factor-gene regulation network

The optimal cross-talk genes with the most intimate relationships were MAPK3 and NFKB1 (Figure 7A), thereby indicating a vital role in the TF-target network.

Figure 7. Protein–protein interaction (PPI) networks and correlation between diagnostic cross-talk genes and infiltrating immune cells. (A) IBD-related TFs chosen from TRRUST and ChEA3, indicated by red circles. The green circles represent the top 50 significant differentially expressed genes in the IBD dataset. Yellow circles indicate the diagnostic 10 cross-talk genes. (B) Gene Ontology pathway enrichment analysis of the 10 cross-talk genes. (C) Correlation between FDX1 and infiltrating immune cells. (D) Correlation between MAPK3 and infiltrating immune cells. (E) Correlation between EPHX2 and infiltrating immune cells. (F) Correlation between NFKB1 and infiltrating immune cells. The degree of the correlation to which the size of the dots indicates genes and immune cells. Correlation strength is proportional to the size of the dots. The color of the dots indicates the *p*-value; a gray color indicates a lower *p*-value, while a yellow hue indicates a higher *p*-value. *p*-Value <0.05 was deemed to be statistically significant.

**Figure:**
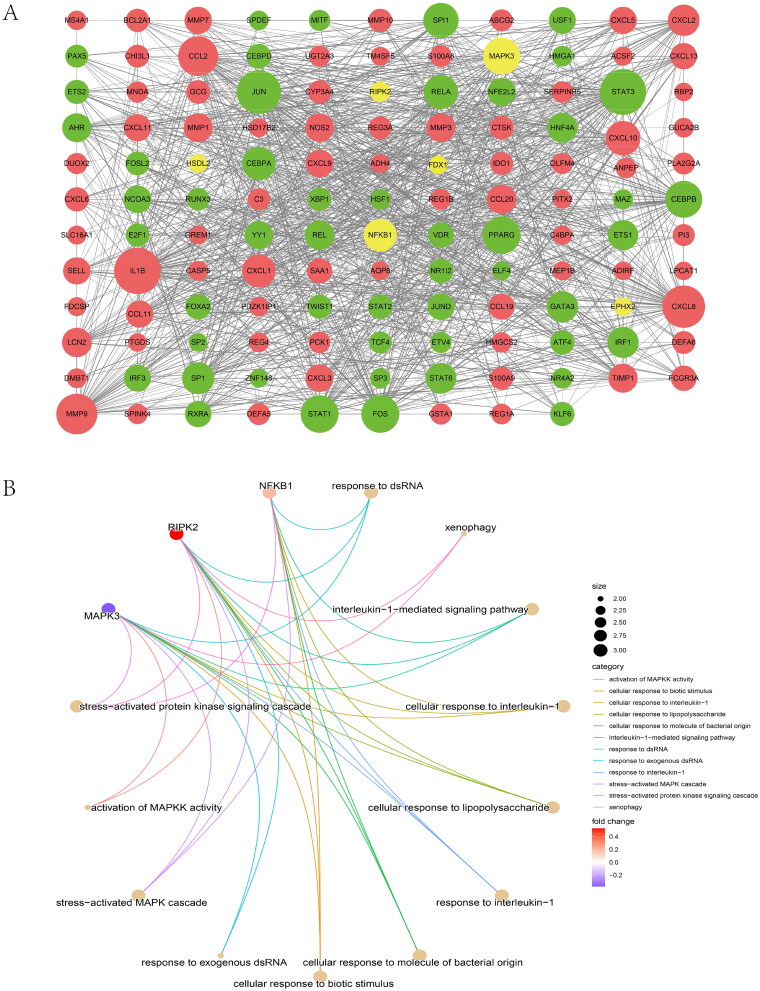

**Figure:**
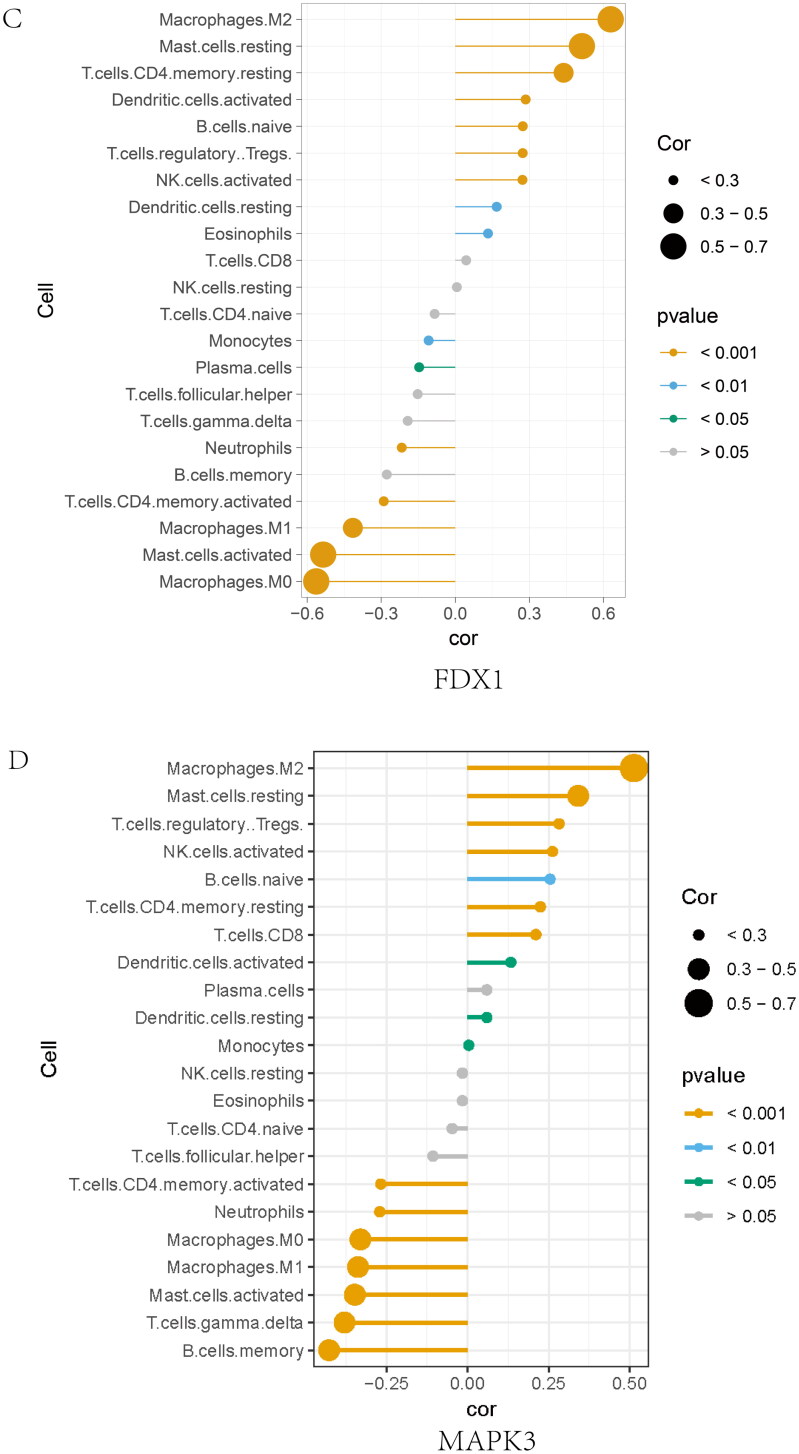

**Figure:**
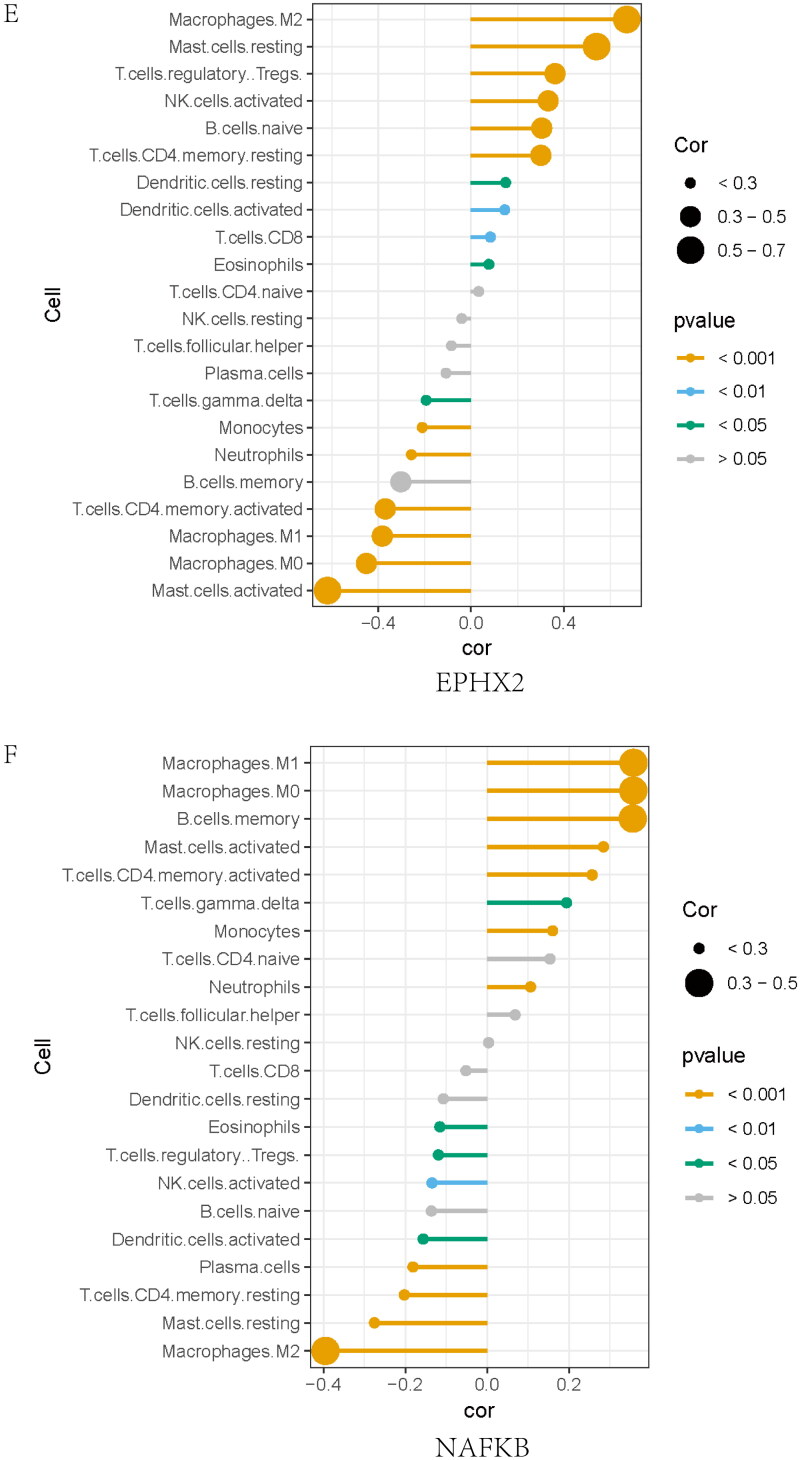

Fifteen crucial pathways were identified (Supplementary Table 6). We established pathway-gene cross-talk between IBD and IgAN to recognize the pathway cross-talk network (Supplementary Figure 6A). Consequently, lipid metabolism was indispensable for the cross-talk between IgAN and IBD. The expression of the 10 genes in the two diseases is shown in Supplementary Figure 6B. Figure 7B shows the GO enrichment of the 10 genes.

### Diagnostic cross-talk genes and immune cells

The CIBERSORTx analysis showed a positive correlation between FDX1 and macrophage M2 (*r* = 0.602, *p* = 2.2E − 16; Figure 7C, Supplementary Figure 7A), but a negative correlation with macrophage M0 (*r* = −0.471, *p* = 2.2E − 16). Interestingly, MAPK3, FDX1, and EPHX2 expression exhibited a similar correlation with these immune cells (Figure 7D and E, Supplementary Figure 7B). MAPK3 expression showed a positive correlation with macrophage M2 (*r* = 0.523, *p* = 2.2E − 16), but a negative correlation with macrophage M0 (*r* = −0.309, *p* = 1.10E − 07). EPHX2 expression exhibited a similar correlation. NFKB1 expression was positively correlation with macrophage M0 (*r* = 0.266, *p* = 9.26E − 07) but negatively correlated with macrophage M2 (*r* = −0.43, *p* = 3.52E − 14) and resting mast cells (*r* = −0.253, *p* = 1.61E − 05) (Figure 7F, Supplementary Figure 7C). These findings are consistent with those of previous studies. Further, NF-κB is implicated in M1 polarization and intercedes pro-inflammatory response in macrophages [46].

### GSEA analysis of cross-talk genes

The single-gene GSEA algorithm was used to determine the potential mechanism of cross-talk between genes in IBD and IgAN (Figure 8A). According to the median cross-talk gene expression status, IBD and IgAN samples converged into high- and low-expression teams. The single-gene GSEA showed that MAPK3 may act on leukocyte migration in IBD and complement activation in IgAN, NFKB1 may affect the inflammatory response in IBD and endocytosis in IgAN, FDX1 may be involved in the adaptive immune response in IBD and cell activation in IgAN, and EPHX2 may function in the innate immune response in IBD and organic acid metabolic processes in IgAN.

Figure 8. Gene set enrichment analysis of the cross-talk genes in IBD and IgAN. (A) The top five gene sets (based on GSEA enrichment score) are presented. (B) Subcellular localization of four cross-talk genes. The cell nucleus was marked blue; the cytoplasm was marked red. The second line is the location of the target protein in the cell, greenish. (The exact url links from Human Protein Atlas was: https://www.proteinatlas.org/ENSG00000137714-FDX1/subcellular#img, https://www.proteinatlas.org/ENSG00000102882-MAPK3/subcellular#img, https://www.proteinatlas.org/ENSG00000109320-NFKB1/subcellular#human). (C) Drug–gene network. The red circles represent four cross-talk genes; the green circles represent underlying drugs interacting with cross-talk genes. (D) Representative histological images of sections stained (with H&E, PAS, and Masson’s trichrome) stain. (E) Immunohistochemical staining results of FDX1, MAPK3, and NFKB1 in kidney tissues (scale bar = 50um). (F) Immunoblotting of the protein expression levels of the FDX1 and NFKB1 in IBD and C56BL/6 mice (The samples derive from the same experiment and that gels were processed in parallel). GAPDH was assayed as an internal control.

**Figure:**
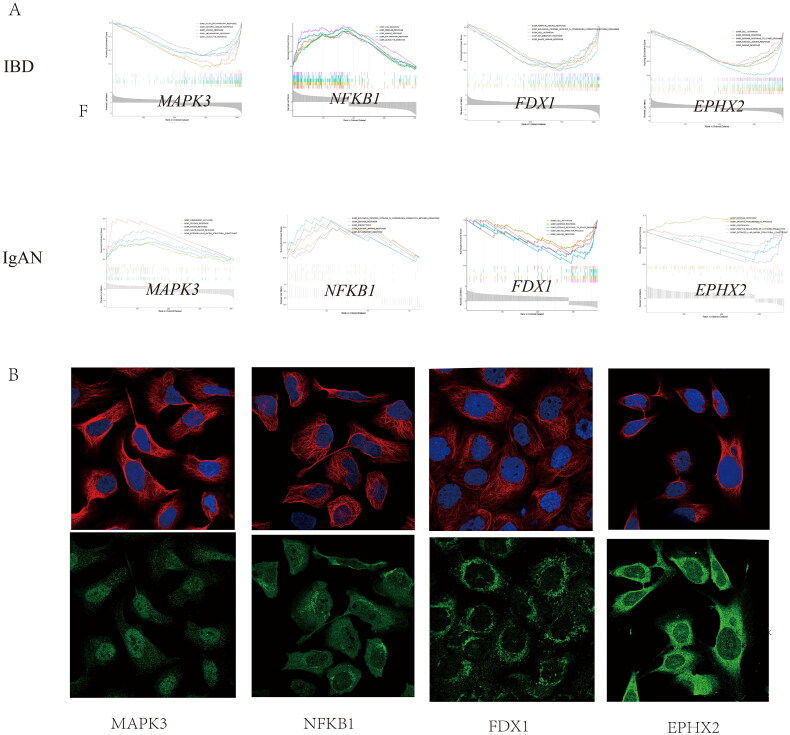

**Figure:**
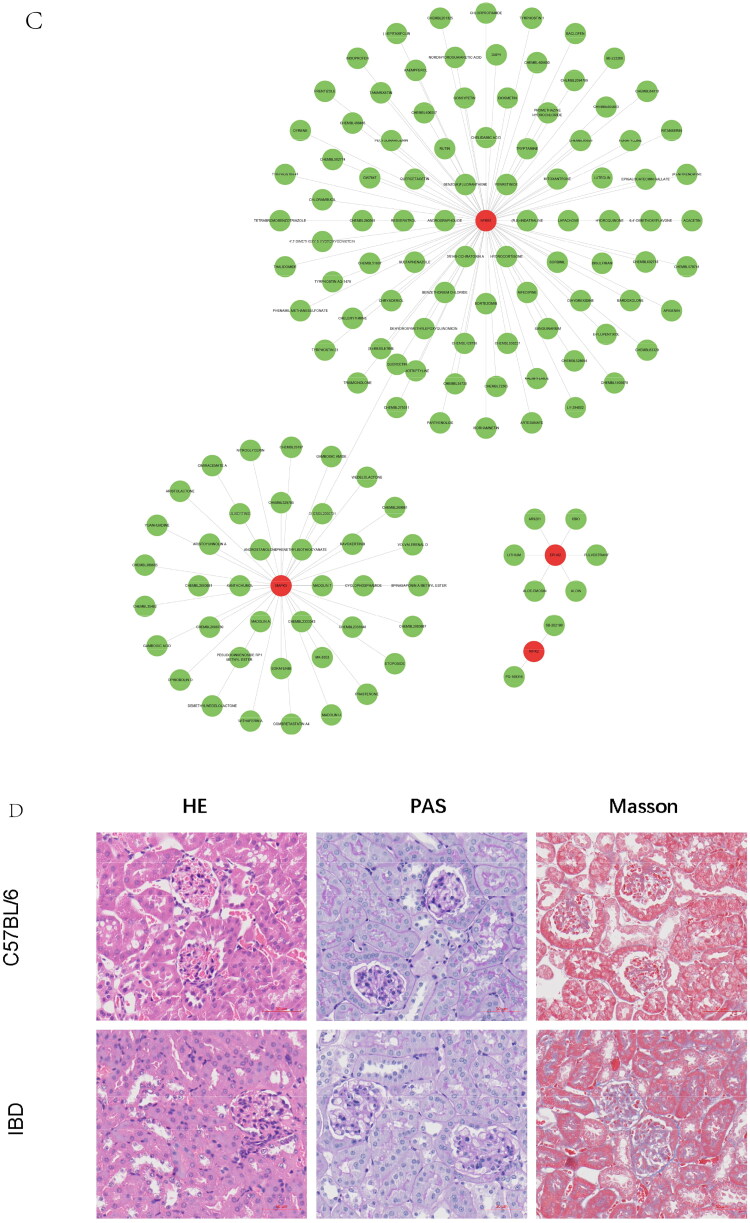

**Figure:**
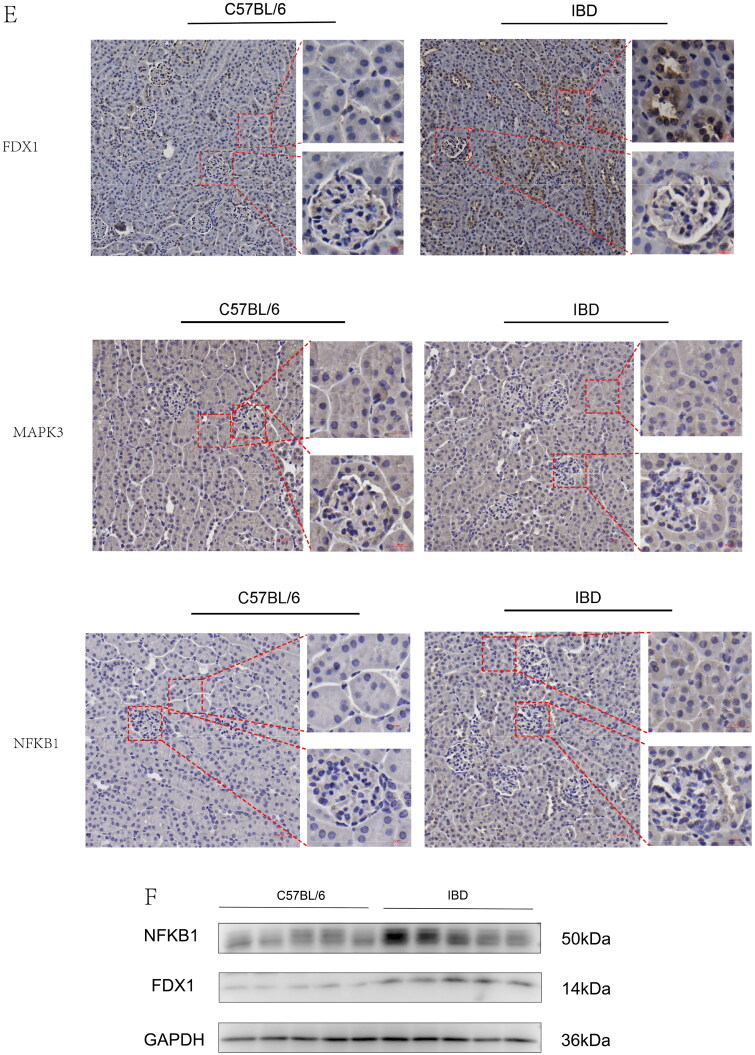

### miRNA–mRNA interaction network

The network analysis tool explored miRNAs as regulatory molecules in diagnostic gene cross-talk. A comparison of the targets of the cross-talk genes showed MAPK3 and NFKB1 to be a potential target of 85 and 79 miRNAs, respectively. Supplementary Figure 6C shows the miRNA-gene regulation network.

The mRNA subcellular localization results revealed that MAPK3, NFKB1, FDX1, and EPHX2 was mainly localized in the nucleoplasm (Figure 8B); nucleoplasm, mitochondria, and cytosol; mitochondria; and cytosol, respectively.

### Recognition of drug–gene interactions

Drug–gene network analysis indicated that 40, 6, 2, and 90 genes were related to MAPK3, EPHX2, RIPK2, and NFKB1, respectively (Figure 8C). These central drugs derived from mutually diagnostic cross-talk genes are fundamental for understanding the mechanism of cross-talk between the genes and function of cells sensitive to receptors [47].

### Activation of cross-talk genes in the kidney of IBD mice

IBD mice exhibited renal histological injury. H&E staining revealed an increased number of cell nuclei in the glomeruli, and periodic acid-Schiff (PAS) staining showed the mesangial matrix and proliferation of mesangial cells. IBD mice exhibited serious glomerular and tubulointerstitial lesions, including mesangial matrix expansion and tubulointerstitial infiltration (Figure 8D). Immunohistochemical staining revealed increased FDX1 and NFKB1 protein levels in the IBD group compared to those in the control group (Figure 8E), thereby suggesting the damaging effect of FDX1 and NFKB1 on kidney injury in IBD mice. However, MAPK3 protein level did not differ significantly between the IBD and control group. We further used immunoblotting to validate the two genes that were found to be elevated in IBD mice using immunohistochemical staining (Figure 8F). Immunoblot analysis revealed that FDX1 and NFKB1 levels were higher in IBD mice than those in C57BL/6 mice, further validating our results.

## Discussion

We report, for the first time, that lipid metabolism, identified *via* enrichment analysis, may represent an essential step for investigating the cross-talk between IgAN and IBD at the transcriptome level. Lipid metabolism is associated with immunological and inflammatory responses [48,49]. Short-chain fatty acids (SCFAs) are significant signaling factors for gut microbiome and intestinal immune system, which contribute to IBD [50–53]. Free fatty acid receptor (FFAR), also called G-protein coupled receptor (GPR) [54,55], has been identified in intestinal endocrine L-cells; FFAR is primarily activated by acetate and propionate [54,56]. Once acetate is activated, FFAR2 promotes and mediates the production of IgA [57], whose deposition in the glomerular mesangium is a feature of IgAN [58]. Notably, the disease activity was associated with reduced SCFAs in IBD [59,60]. SCFAs alleviate the clinical symptoms and pathological damage related to IgAN [61], and intestinal fatty acid levels are remarkably decreased in patients with IgA [62,63]. These compelling findings indicate a latent function of lipid metabolism in the link between IBD and IgAN.

Other significant pathways identified by Kyoto Encyclopedia of Genes and Genomes analysis, such as glycolysis, are vital in IBD [64]. The energy and metabolites produced by glycolysis support the development and metabolism of bowel microbiota [65]. The glycolytic metabolic pathway regulates the immune system [66]. Similar to lipid metabolism, glycolysis can adjust the macrophage activation state and cell function by regulating polarization [67,68]. However, the association between glycolysis and IgAN remains poorly understood. Further investigation into this may provide a feasible treatment for IgAN.

Dharmasiri et al. reported, for the first time, intestinal macrophages (M1 and M2) in patients with IBD [69]. Their data supported the idea that M2 macrophage exacerbates M2-associated Crohn’s disease-specific characteristics, including fibrosis and granuloma formation. Therefore, M2 macrophage may be involved in IBD progression. Efforts are ongoing to clarify the role of M2 macrophage in IBD pathogenesis. Yang et al. observed significantly increased M2 macrophage infiltration in the renal tissues of patients with IgAN [70]. In patients with IgAN, M2 macrophage is positively correlated with 24-h proteinuria and serum creatinine [70]. This study demonstrates that M2 macrophage is essential in the evolution of IgAN fibrosis. M2b macrophage is one of the predominant subpopulations in the kidney tissues of patients with IgAN [71]. These findings highlight the role of neutrophils and macrophages in the immunological mechanisms underlying IBD and IgAN.

The significant cross-talk gene NFKB1 is a member of the nuclear factor kappa B (NF-κB) family that encodes p50. In multiple biological processes, including inflammation, immunity, cell growth, apoptosis, and differentiation, NFKB1 is implicated as a transcription factor [72]. A correlation between inflammation and renal damage has been observed during IgAN progression [73]. Han et al. supported this association with inflammation based on the evidence that high NFKB activity in the bowel reflects a high inflammatory burden [74]. A function genomics screen showed that NFKB1 regulated the expression of S100A8 and S100A9, which are the subunits of CP secreted by macrophages and neutrophils, a critical IBD biomarker for monitoring disease severity [75–77]. In addition, macrophages infiltrate the intestinal mucosa of patients with IBD and are deemed crucial for pathology, producing inflammatory mediators, such as TNF-α [78]. Interestingly, glomerular visceral epithelial cells can also produce TNF-α, which plays a significant role in an aberrant mucosal immune response leading to glomerulonephritis [79]. NF-κB signaling is implicated in proteinuria and podocyte damage [80,81]; and a crucial step in IgAN before ESRD development is the immune-sensing inflammation by NF-κB activation [82]. The NF-κB signaling pathway was activated in model rats with IgAN [83]. Our findings highlight the importance of NFKB1 in IBD and IgAN progression. Here, we show that the abnormal physiological processes of IBD and IgAN are associated with NFKB1 and MAPK3 expression (Figures 5A and 7A). Another cross-talk gene identified in this study was MAPK3. The GO results enriched 10 cross-talk genes in the MAPK family pathway (Figure 5C). Although the MAPK3 protein level in IBD mice was not significantly different from that in the control group (as evidenced *via* immunohistochemical staining), previous studies have associated MAPK3 with IBD and IgAN [76,79,80]. Therefore, further research is warranted to explore this.

We obtained kidney tissue from IBD mice and observed elevated FDX1 expression, which is particularly vital for advocating our proposed viewpoint. FDX1 is a newly discovered gene that regulates cuprotosis [84–86]. Cuprotosis, a mode of programmed cell death, was recently recognized by TODD R. GOLUB’s team in 2022 and occurs through the direct combination of copper with lipid-acylated components of the TCA cycle, resulting in the clustering of lipid-acylated proteins and reduction of iron–sulfur cluster proteins, thereby leading to proteotoxic stress and ultimately cell death [87,88]. The effect of FDX1 expression on various diseases, such as clear cell carcinoma, colon adenocarcinoma, and polycystic ovary syndrome, has aroused broad concern [84,85,89,90]. Xiao et al. delineated a scoring mechanism, the cuprotosis-mediated pattern-related prognostic (CMPRG_score) score [91], and evaluated the correlation between the score and immune cell abundance using the CIBERSORTx algorithm. Surprisingly, the CMPRG_score was significantly positively correlated to M2 macrophage and neutrophils [91]. The potential relationship between FDX1 and IgAN was first reported seven years ago [92,93]. We present an innovative perspective that explains cuprotosis and paves the way for exploring the potential link between IgAN and IBD.

In addition to the critical genes mentioned in this study, more than a decade ago, researchers proposed that the HLA-DR1 allele in IgAN and HLA-DR1/DQw5 allele in Crohn’s disease form a mutual genetic basis for the two diseases [94]. Moreover, using genome-wide association studies, Shi et al. identified CFB, a shared risk locus for the two diseases, that functions in complement activation [95]. The search for genes susceptible to both diseases is ongoing [96–98], and our study contributes to the exploration of its potential role. Regarding the prospects in clinical therapeutics, our findings can serve as novel diagnostic biomarkers and clinical treatment guidance for patients with IBD and IgAN. Focusing on these genes when IBD and IgAN co-occur may help to identify intervention targets that can treat both diseases. Furthermore, the filtration of IgAN and identification of early signs of kidney damage in patients with IBD can provide critical information on the mechanisms responsible for minimizing or reversing further renal deterioration. In this exploratory study on the application of bioinformatics analysis to identify the potential cross-talk between IBD and IgAN, we demonstrated similar immunoinvasive microenvironments and cross-talk genes. Altogether, our study identified crucial cross-talk genes and several shared pathways, thereby emphasizing the comparability and latent relationships between IBD and IgAN. Patients with IBD may develop kidney damage caused by lipid metabolism disorders. To this end, more efforts are needed to elucidate the underlying mechanism between these two diseases.

## Conclusion

In summary, a comprehensive bioinformatics analysis was used to explore and validate the diagnostic cross-talk of genes associated with IBD and IgAN. Our results revealed that immune cells (M2 macrophage and neutrophils) interact with the cross-talk genes. The cross-talk genes, immune cells, and common pathways in IBD and IgAN were correlated. These insights may provide opportunities for the development of novel therapeutic strategies.

## Supplementary Material

Supplemental Material

## Data Availability

The code and data can be found on GitHub at the following link: https://github.com/qqyxasdq/Novel-Immune-Cross-talk-Between-IBD-and-IgAN.git
