# Supplementary material for: Novel immune cross-talk between inflammatory bowel disease and IgA nephropathy
Source: Ren Fail. 2024 Apr 17;46(1):2337288. doi: 10.1080/0886022X.2024.2337288 (PMC11025414; doi:10.1080/0886022X.2024.2337288)

Supplementary tables:

Supplementary table 1: The IBD patients' specific biopsy site

Biopsy site	Number of samples
ascendens	33
bulbus durodenum	15
coecum	23
descendens	53
ileum	39
rectum	70
sigmoideum	87
transversum	42
valvula	1
uncertain	5

Supplementary table 2: Definitive diagnosis of the sample

Patient type	Number of samples
CD	103
UC	161
Control	99
uncertain	5

Supplementary table 3: R packages corresponding to the algorithm

Algorithm	R packages
GSVA algorithm	"GSVA"、"GSEABase"
ssGSEA	"tidyverse"、"GSVA"、
xCell	"Xcell"
WGCNA	"WGCNA"、 "DESeq2"
LASSO regression	"glmnet"
RFE algorithm	"caret"、"survival"
Boruta algorithm	"Boruta"
SVM model	"e1071"、"caret"
RF model	"randomForest"、 "caret"、 "Proc"
GLM model	"lme4"
spearman's rank correlation test	"ggplot2"、"dylyr"、"linkET"
single-gene GSEA algorithm	"clusterProfiler"、"org.Hs.eg.db"

Supplementary table 4: Top 10 in network 40 genes ranked by MCC method

Rank	Name	Score
1	MAPK3	288
2	CD19	230
3	TLR10	180
4	CAT	174
5	NAKB1	164
6	BLK	102
7	ABCB1	84
8	MS4A1	59
9	CCL19	58
10	CCL21	56

Supplementary table 5: Selection of feature genes by three algorithms

Boruta	REF	LASSO
BID	EPHX2	ABCB1
ASNS	MAPK3	NID2
ST6GAL1	RIPK2	PGRMC1
ABCB1	KDF1	MAPK3
DNAJA1	PCK2	RIPK2
PCK2	RIDA	NFKB1
PGRMC1	HSDL2	HSDL2
MAPK3	SYNPO	EPHX2
RIPK2	METTL7A	RIDA
NFKB1	NFKB1	FDX1
HSDL2	BID	WDR20
EPHX2	FDX1	SYNPO
CAT	GPX2	KDF1
RIDA	PLA2R1	KNTC1
FDX1	DEPTOR	METTL7A
SLC3A1	HPGD	CD37
WDR20		CCL21
PLA2R1		
DEPTOR		
HPGD		
SYNPO		

KDF1
GPX2
METTL7A
MAP3K5
BLK
CCL21
MS4A1
CCL19

Supplementary table 6: 15 crucial pathways function in both diseases.

ID	Pathway
hsa00010	Glycolysis / Gluconeogenesis
hsa00071	Fatty acid degradation
hsa00020	Citrate cycle (TCA cycle)
hsa00030	Pentose phosphate pathway
hsa00120	Primary bile acid biosynthesis
hsa00052	Galactose metabolism
hsa00100	Steroid biosynthesis
hsa00061	Fatty acid biosynthesis
hsa00130	Ubiquinone and other terpenoid-quinone biosynthesis
hsa00051	Fructose and mannose metabolism
hsa00062	Fatty acid elongation
hsa00190	Oxidative phosphorylation
hsa00053	Ascorbate and aldarate metabolism
hsa00140	Steroid hormone biosynthesis
hsa00040	Pentose and glucuronate interconversions

Supplementary Figures:

Supplementary Figure1:

A

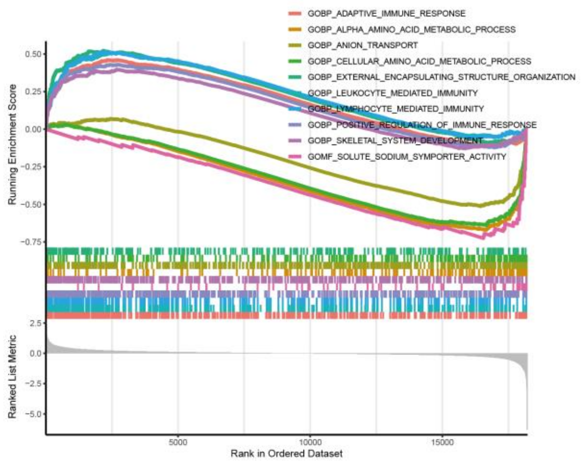

B

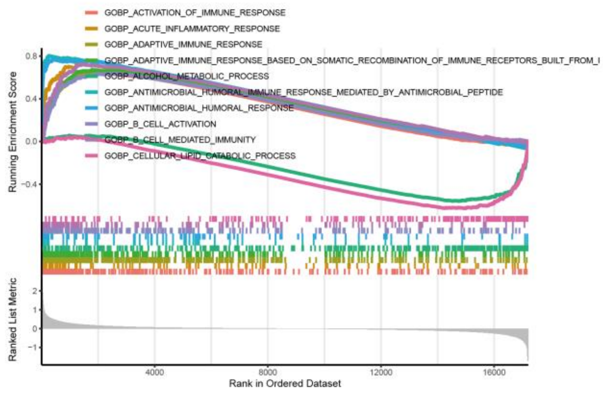

Supplementary Figure 1. The expression level of all DEGs.

The expression level of all DEGs in IBD dataset (A) and IgAN dataset (B).

Supplementary Figure2:

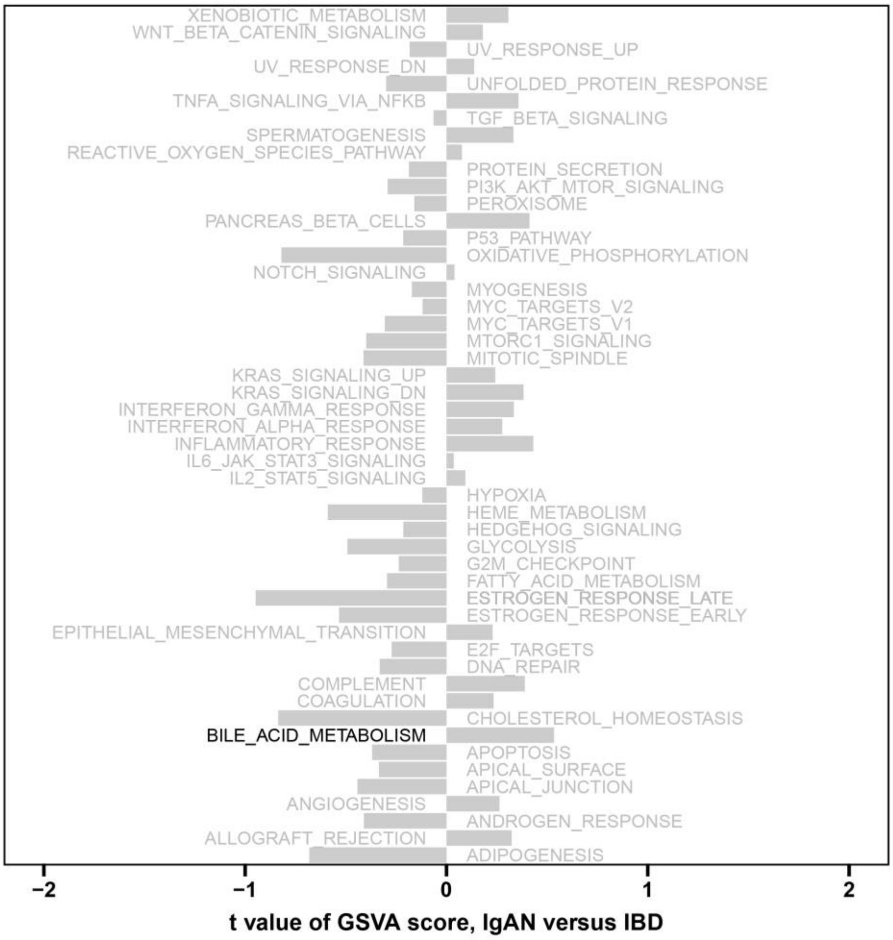

Supplementary Figure 2. The underlying immune characteristics diversity between IgAN and IBD samples.

The differences of the Reactome pathway enrichment score between IgAN and IBD samples.

Supplementary Figure3:

A

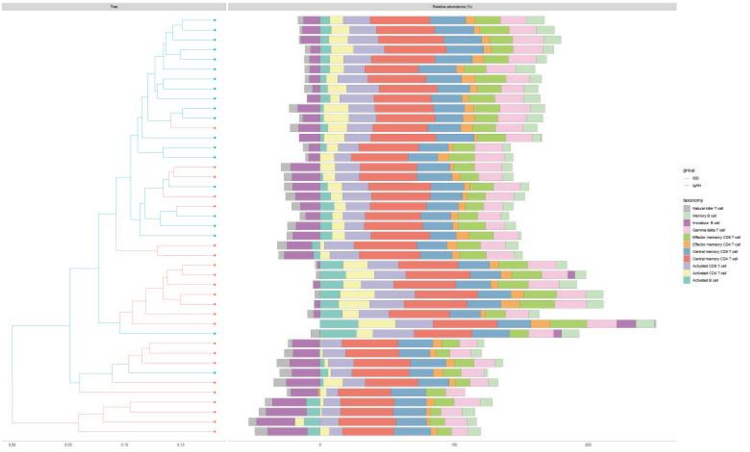

B

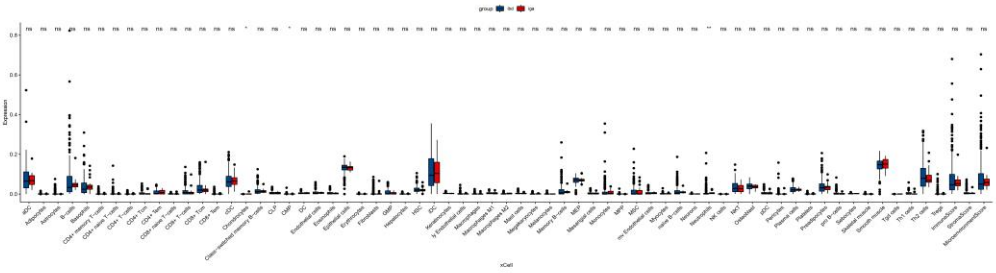

C

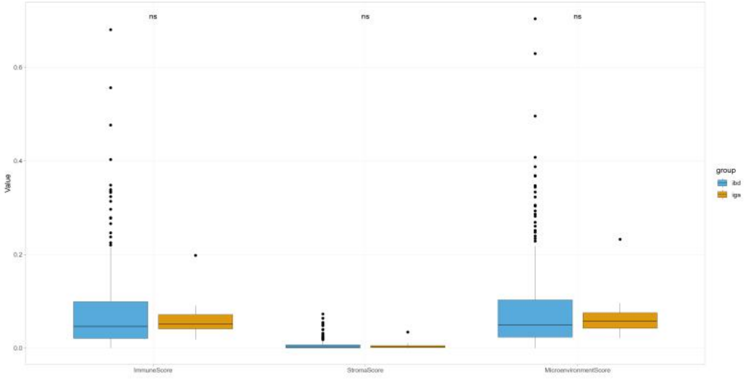
ns
ns
ns
0.6
0.4
0.2
0.0
Value
group
ibd
iga
ImmuneScore
StromaScore
MicroenvironmentScore

Supplementary Figure 3. Comparison of the immune cells between IBD and IgAN samples.

(A)An intuitive picture of the percentage of the ssGSEA (11 immune cells) in IBD patients and IgAN patients. (B, C) Comparison of the scores for immune cells estimated by xCell algorithm between IBD and IgAN samples. (*P < 0.05, **P < 0.01; Mann-Whitney U test). ns, not significant.

Supplementary Figure4:

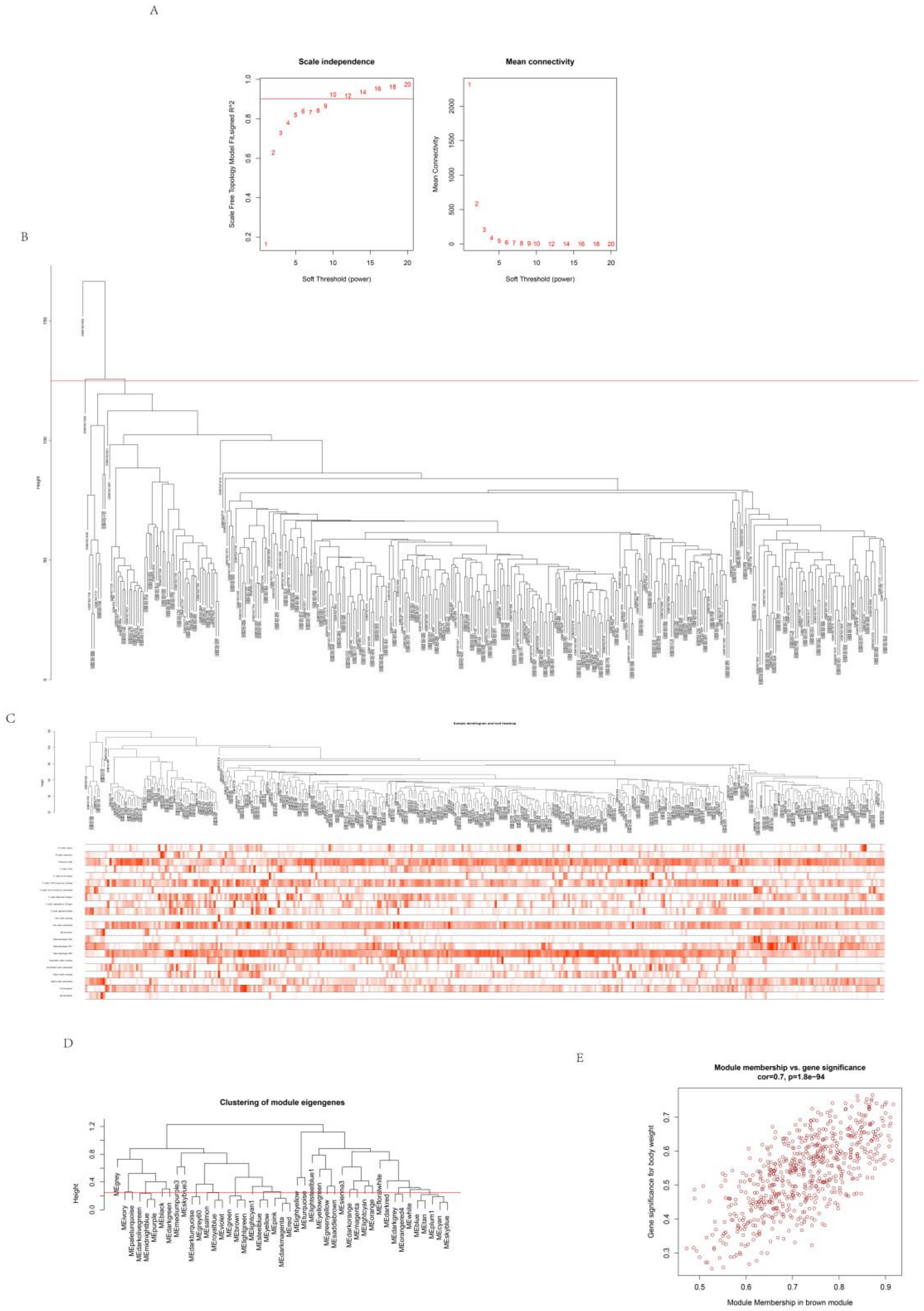

Supplementary Figure 4. A series of clustering dendrograms for IBD samples.

(A) Scale-free fitting index analysis and mean connectivity of soft threshold power from 1 to 20. **(B)** Sample clustering to detect outliers. **(C)** Sample dendrogram and trait heatmap. **(D)** Clustering of module eigengenes. **(E)** Scatter plot of M2 macrophages in the brown module. In the brown module, GS and MM reveal a very significant correlation, manifesting that the genes of the brown module are highly related to the M2 macrophage module.

Supplementary Figure5:

A

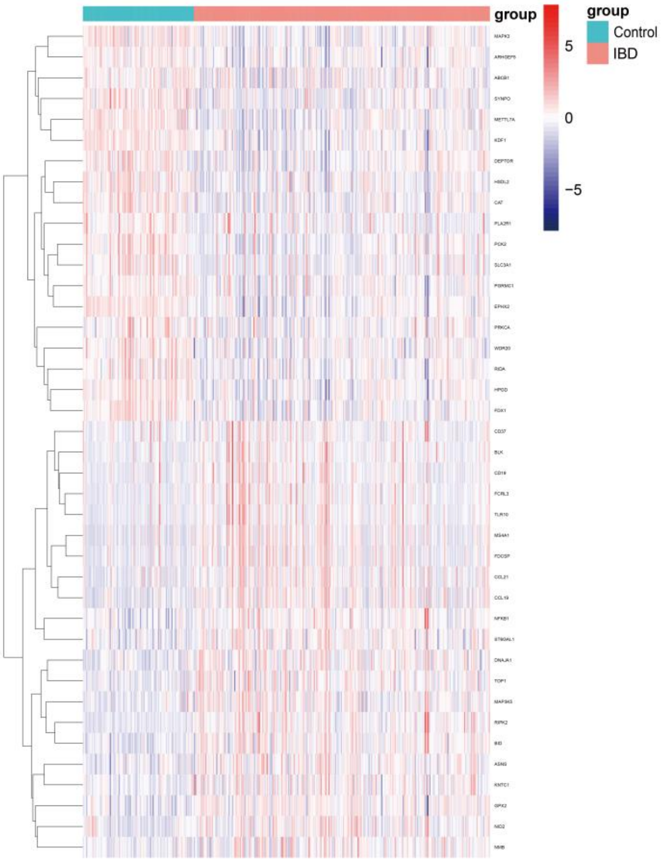

B

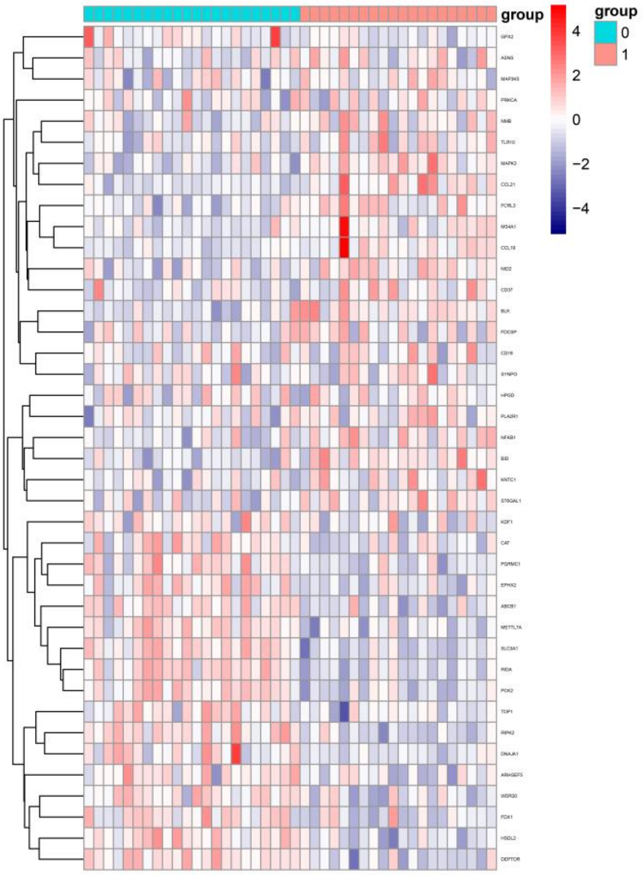

C

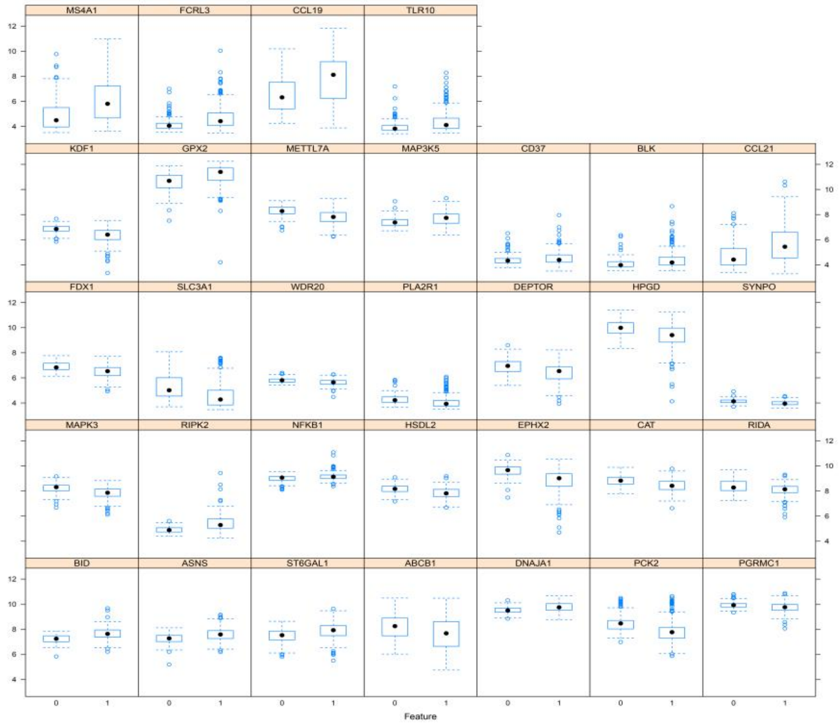
MS4A1
FCRL3
CCL19
TLR10
KDF1
GPX2
METTL7A
MAP3K5
CD37
BLK
CCL21
FDX1
SLC3A1
WDR20
PLA2R1
DEPTOR
HPGD
SYNPO
MAPK3
RIPK2
NFKB1
HSDL2
EPHX2
CAT
RIDA
BID
ASNS
ST6GAL1
ABCB1
DNAJA1
PCK2
PGRMC1
Feature

D

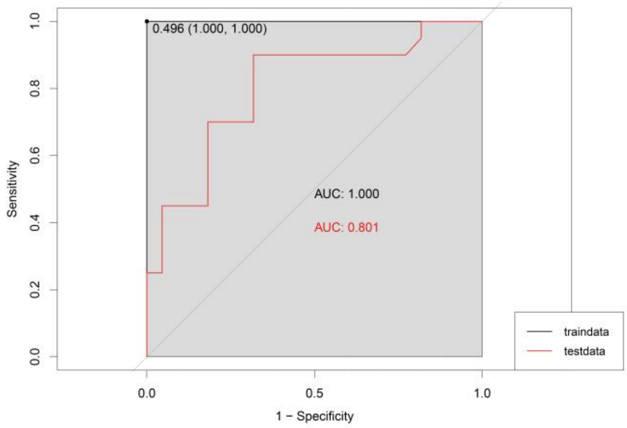
0.496 (1.000, 1.000)
AUC: 1.000
AUC: 0.801
Sensitivity
1 − Specificity
traindata
testdata

E

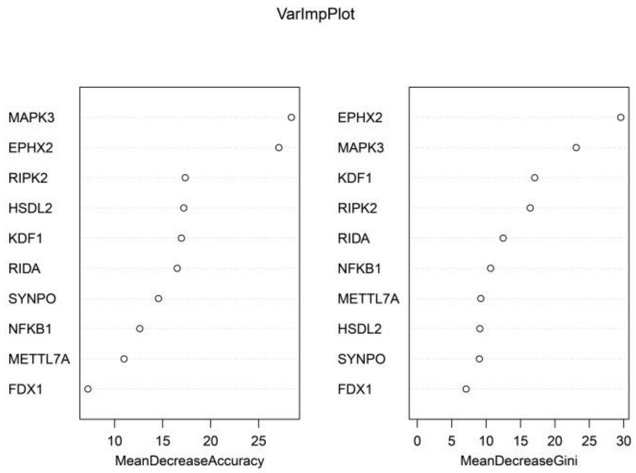
VarImpPlot
MAPK3
EPHX2
RIPK2
HSDL2
KDF1
RIDA
SYNPO
NFKB1
METTL7A
FDX1
10 15 20 25
MeanDecreaseAccuracy
EPHX2
MAPK3
KDF1
RIPK2
RIDA
NFKB1
METTL7A
HSDL2
SYNPO
FDX1
0 5 10 15 20 25 30
MeanDecreaseGini

F

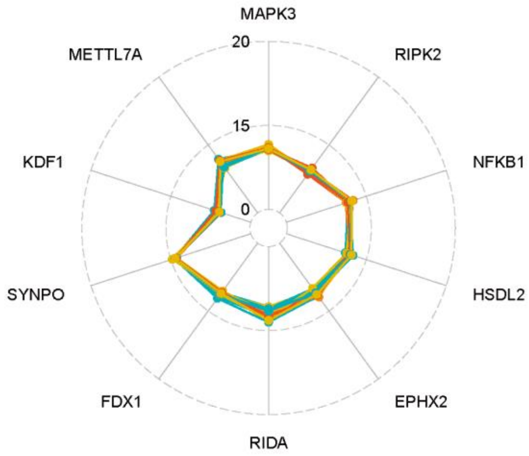
MAPK3
RIPK2
NFKB1
HSDL2
EPHX2
RIDA
FDX1
SYNPO
KDF1
METTL7A
20
15
0

G

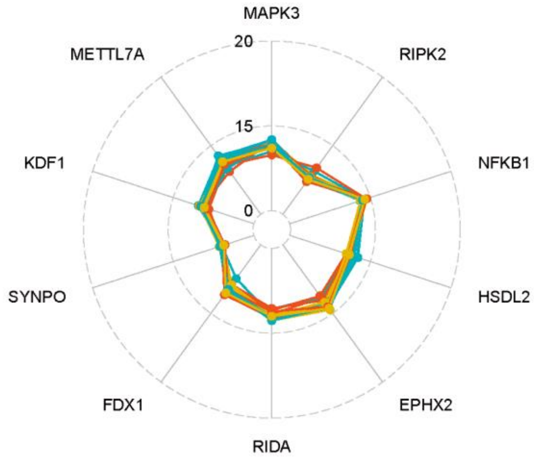
MAPK3
RIPK2
NFKB1
HSDL2
EPHX2
RIDA
FDX1
SYNPO
KDF1
METTL7A
20
15
0

Supplementary Figure 5. The expression level of 40 common genes in the IBD dataset **(A)** and IgAN dataset **(B)**. (C)**The expression of the features selected by the Boruta algorithm in the IBD dataset (IBD samples vs healthy controls). (D)** The receiver operating characteristic (ROC) curves of the RF model. **(E)** Importance ranking based on the RF model of the 10 cross-talk genes. The Raders chart shows the expression of the 10 cross-talk genes in IBD **(F)** and IgAN patients **(G)**.

Supplementary Figure6:

A

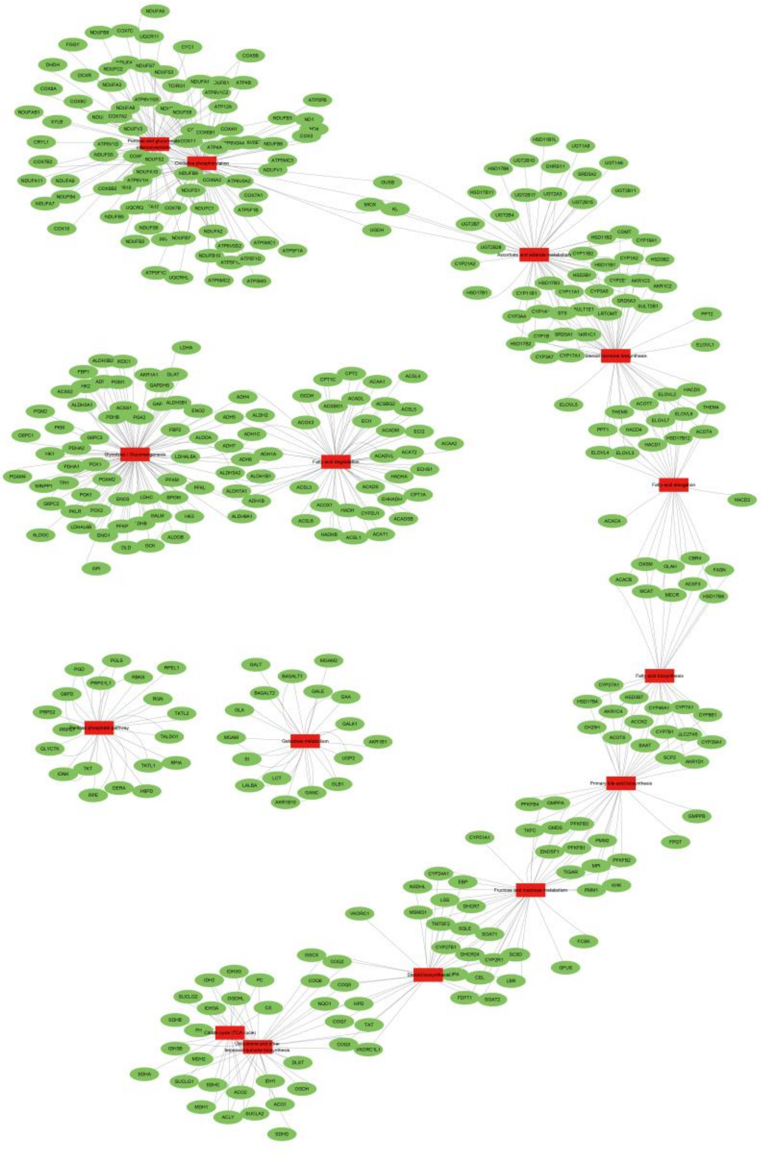

B

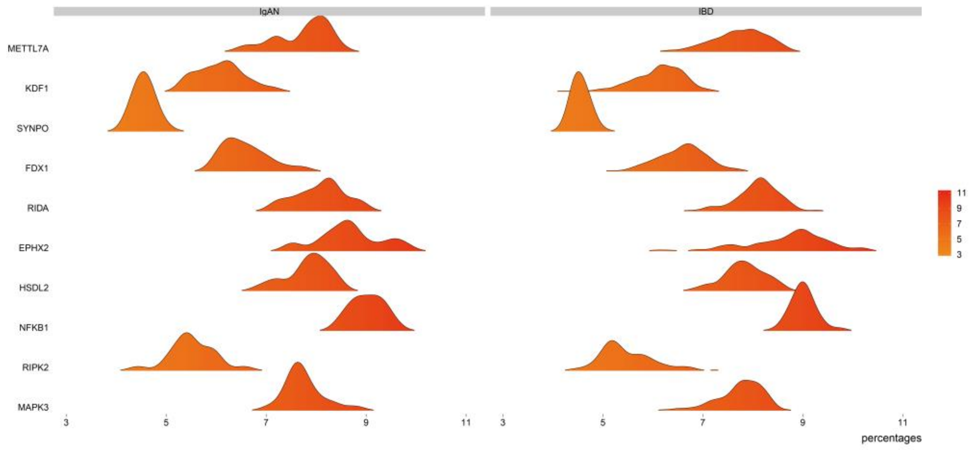

C

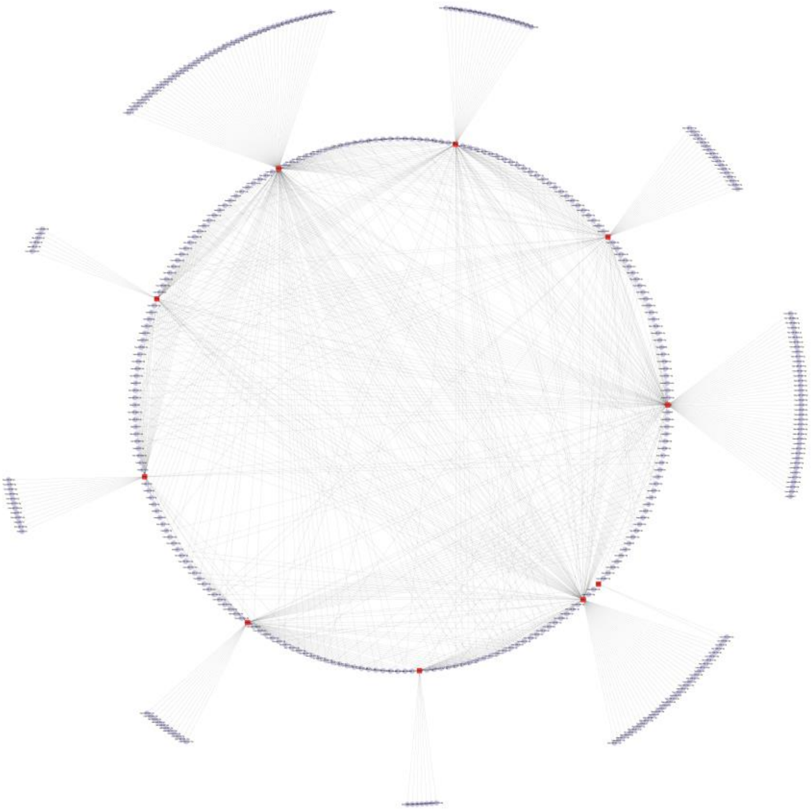

Supplementary Figure 6.

(A) Fifteen crucial pathways functioning in both IBD and IgAN, indicated by red squares. The green circles represent the genes from each pathway, both IBD-related

and IgAN-related. **(B)**The ridgeline plots showed the density distribution of the 10 cross-talk genes in the IBD and IgAN datasets. **(C) miRNA- mRNA network.** The red squares represent ten diagnostic cross-talk genes; the purple circles represent miRNAs associated with the cross-talk genes.

Supplementary Figure7:

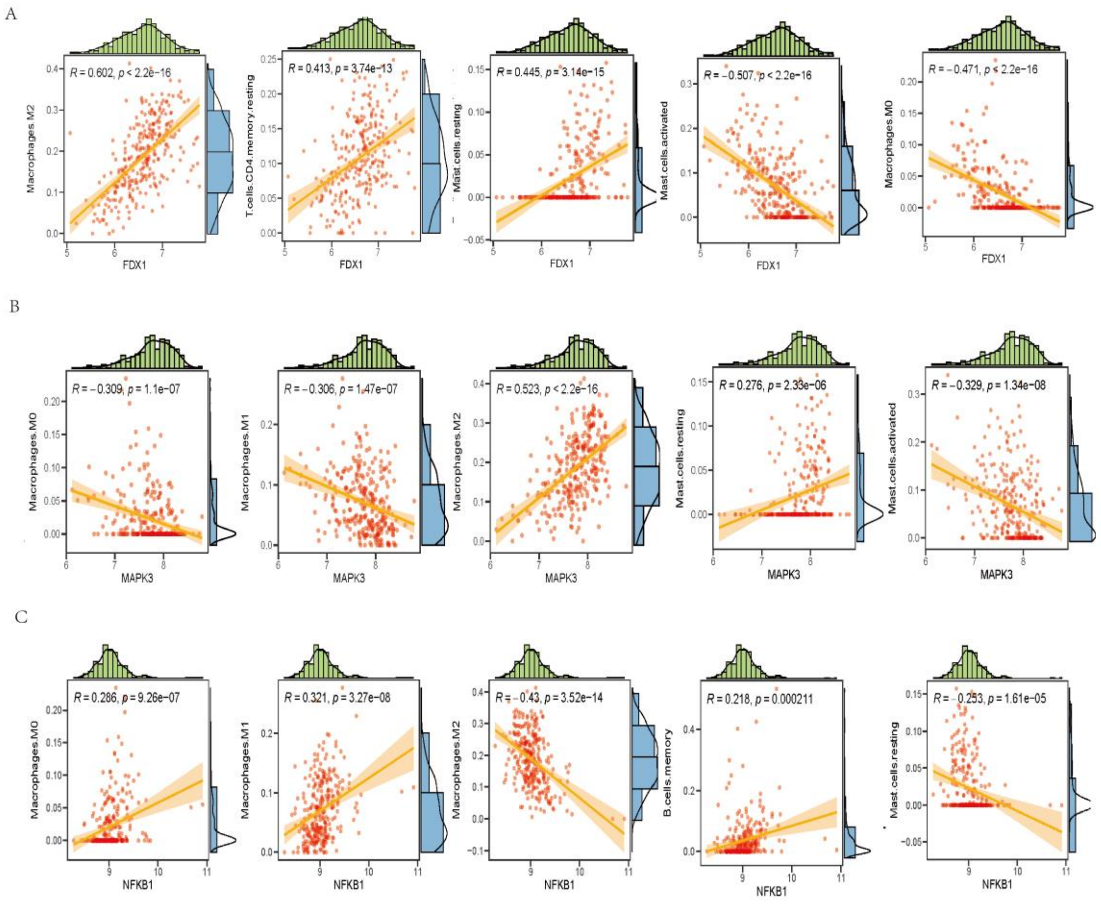

Supplementary Figure 7. Relationship between corss-talk genes and immune infiltration cells.

Supplementary Figure 8. Original blots of Figure 8F.

GAPDH:

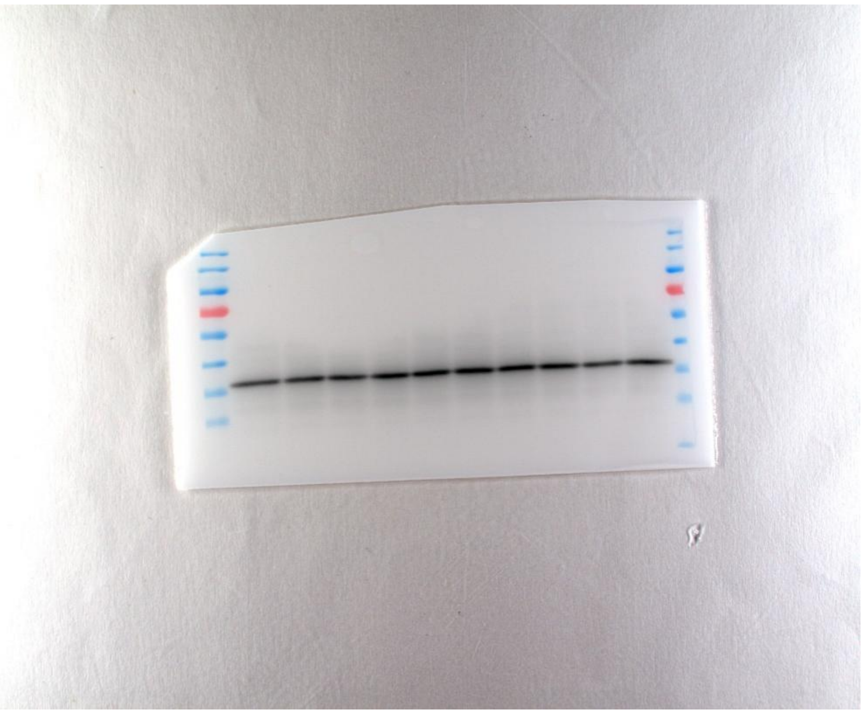

FDX1: (The blot was cut prior to hybridization with antibodies during blotting to improve the clarity.)

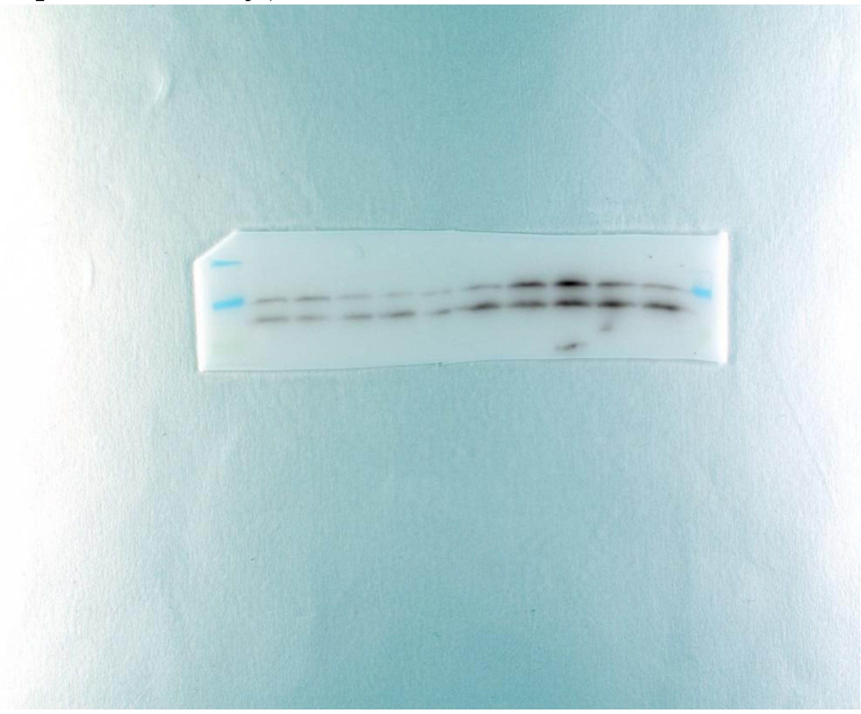

NFKB1:

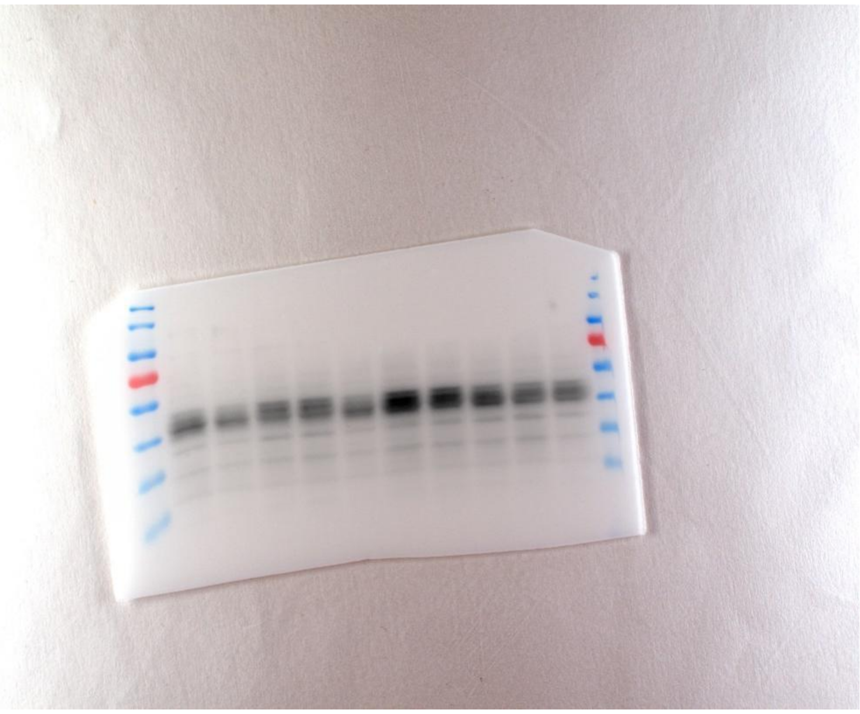

Supplement: Supplemental Material [file IRNF_A_2337288_SM2605.pdf]
